# Study on uneven settlement and correction of steel frame structures based on numerical simulation method

**DOI:** 10.1371/journal.pone.0303249

**Published:** 2024-05-24

**Authors:** Hang Chen, Yuxu Guo, Shagea Alqawzai, Xiaosong Wu

**Affiliations:** 1 School of Civil Engineering, Guang’an Vocational & Technical College, Guang’an, PR China; 2 School of Civil Engineering, Chongqing University, Chongqing, PR China; 3 Department of Engineering Construction, Dazhou Central Hospital, Dazhou, PR China; Jamia Millia Islamia, INDIA

## Abstract

Lifting-correction is a technique to restore buildings experiencing uneven settlement, while ensuring the safety and integrity of the main structural system. This study was based on a real light-steel building structure and provided a detailed description of scenarios involving uneven settlement and the process of lifting and correction. Additionally, a sophisticated finite element (FE) model was established using the generic FE software ABAQUS, with refined material constitutive models to ensure the accuracy of simulation results. Firstly, the impact of uneven settlement on the structure was examined, including modal and stress field analyses. Different methods of breaking column (BC) and lifting column (LC) were compared and scrutinized to identify optimal approaches and minimize damage and disturbance to the building. Four methods have been proposed and compared, including simultaneously breaking columns, breaking columns with chessboard style, simultaneously lifting columns and lifting columns in multiple stages. The four methods were comprehensively evaluated from the perspectives of stress fields, displacement responses, damage and energy dissipation. The results indicated that after uneven settlement, the eigenvalues and frequencies of the structure decrease, the structure tended to be unstable. Simultaneously, as stress increases, some joints’ materials enter the yielding stage, affecting the overall structural stability and safety. When damage occurs in some joints, the structural safety was compromised. The comparison between the two BC methods, including the chessboard style and simultaneously BC methods, it was revealed that the former causes less disturbance to structural initial stress field. The comparison between the two LC methods, including, simultaneously and LC in multiple stages, it was revealed that the latter performs slightly better in terms of stress fields, displacement fields, damage, energy dissipation and internal forces. Therefore, the methods of BC in chessboard style and LC in multiple stages were recommended to use in engineering practice to ensure less structural disturbance. The findings obtained from this study can provide guidance for structural engineers to solve the uneven settlement of buildings.

## 1. Introduction

Building correction/retrofitting refers to the corrective and strengthening measures taken to restore the normal use function of existing buildings that have been occurred due to local vertical displacement affected by local human or natural reasons, which seriously affects the use of building and even endangers the lives and property of residents as well as the factory production safety [1]. Except for major defects that cannot be restored due to design, reasonable technical measures can be taken to make it usable again. Demolition and reconstruction not only consume a large amount of manpower and material resources, causing huge economic losses, but also are difficult to achieve for ancient buildings with cultural and historical values.

The inclination of buildings is primarily a response to the uneven settlement of the foundation, leading to the structural inclination of the building. There are various reasons for uneven settlement of buildings, including factors related to the upper structure, foundation of the building, environmental effects and external factors, as detailed in Table 1. The potential reasons and further explanations of uneven settlement of buildings have been provided in Table 1.

**Table 1:** The reason of uneven settlement of buildings [2].

Summary of the main reasons	Detailed description of the potential reasons of uneven settlement
Affected by upper structure	(1) Asymmetry in building structure and differences in load distribution; (2) Complex building forms and improper layout; (3) Influences during the construction process;
Affected by foundation	(1) Improper foundation treatment and design; (2) Spatial variations in soil layer distribution; (3) Special types of soils (expansive soils, collapsible loess, frozen soil, etc.); (4) Karst, soil caves, landslides, collapses and vibration liquefaction, etc.;
Affected by environmental and external factors interference	(1) Effects of adjacent buildings; (2) Influence of nearby construction on soil layers; (3) Structural inclination caused by natural disasters such as wind forces, earthquakes, etc.

Currently, scholars have proposed various methods for correcting buildings that are subjected to an uneven settlement [3]. The commonly used approaches can be divided into two categories: the first involves lowering the elevations of non-settlement areas to match those of the settlement areas, and the second involves lifting the settlement areas to restore reasonable elevations [4, 5]. The iconic example of corrected inclination is the Leaning Tower of Pisa. By causing the non-settlement area to settle to the same elevation as the settlement area, the ultimate goal of correcting the tilt is achieved [5, 6]. The injection of consolidants and chemical materials into the lower layers, reinforcing the settlement area, is widely applied by engineers in practical construction [7]. In the context of contemporary urban environments, the jacking-up method proves to be more applicable for correcting uneven settlement in buildings, because this method has a smaller impact on surrounding structures and the environment. Jacks are used to lift the settlement area of a building. Displacement and load control can be selected for precise control [8]. Jacks are placed at the base of lower columns after the columns in the settlement area have been truncated. This method has been applied to the correction of inclination in many buildings [9–12].

Research on uneven settlement mainly focuses on special structures, such as pipelines [13, 14], bridges [15–17], tunnels [18, 19], high-speed railways [20], or highways [21]. There is also relevant research examining the uneven settlement of building structures. However, most of these studies on the uneven settlement of building structures primarily concentrate on foundations [22] and soil layers [23, 24]. Limited systematic research has been conducted on the deformation and mechanical responses of superstructures during uneven settlement processes [25]. Feasibility studies on various commonly used uplifting and strengthening methods have not been retrieved. Such research is crucial for practical engineering, as it can directly guide engineers in correcting building structures.

In summary, the correction and strengthening techniques of buildings have been applied in engineering practices by structural engineers. However, there is relatively little research systematically examining the impact of settlement, column truncation, jacking schemes and building correction techniques. To reduce the risks during the jacking process and minimize disturbance to the original structures, the rationality and reliability of jacking schemes need further examination. This paper aims to utilize the finite element (FE) numerical simulation techniques to examine jacking schemes before correcting buildings and investigate the damage caused by the correction. Simultaneously, it analyzes the structural disturbances during uneven settlement stages. Methods of column truncation, jacking, and additional internal force are also investigated. Based on this research, guidance can be provided to structural engineers for dealing with uneven settlement in buildings and correcting building inclinations in engineering practices.

## 2. Technology roadmap

**Fig 1:**
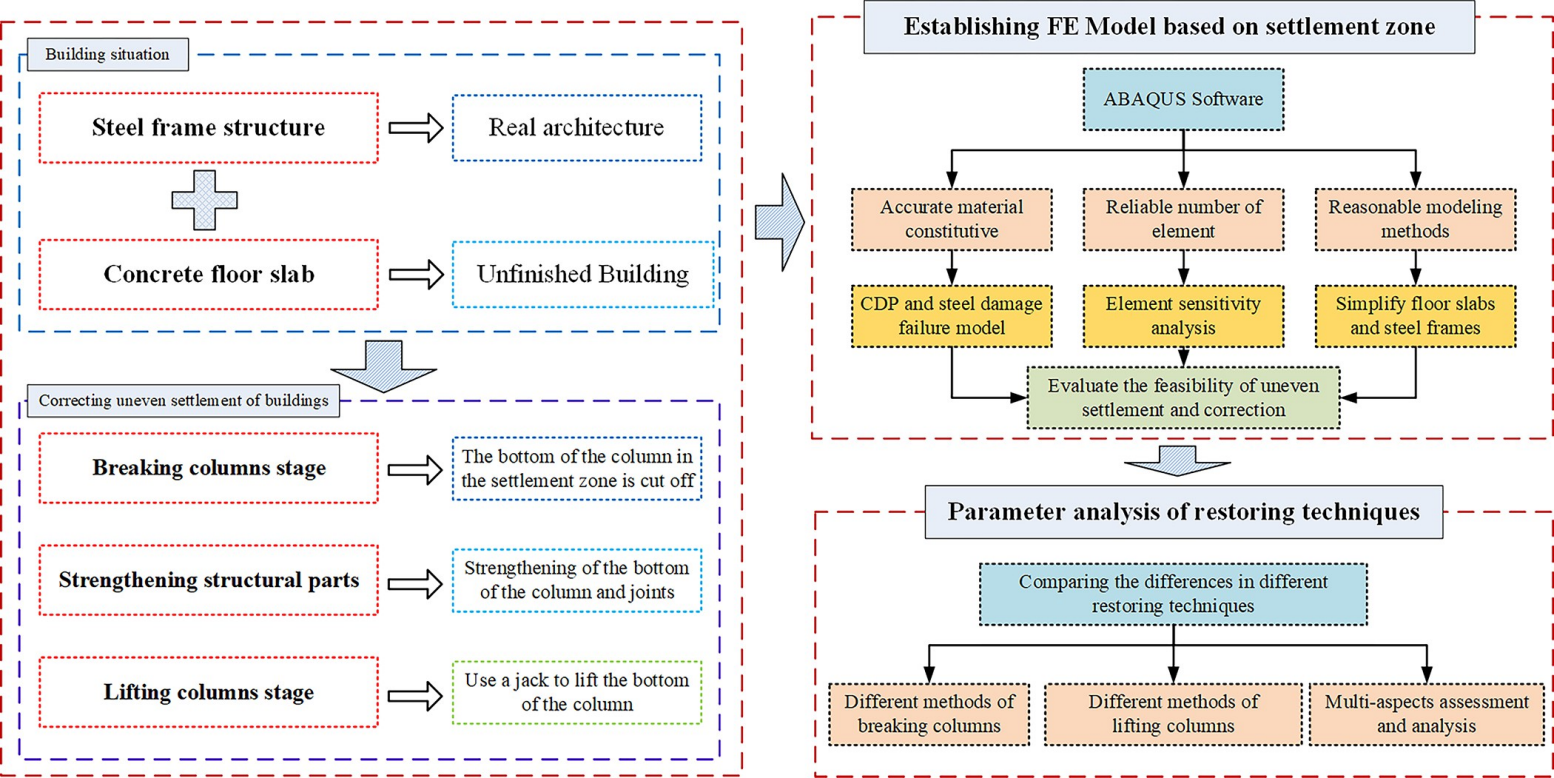
Research technical roadmap.

This study was based on an actual correction and strengthening project for a light steel structure building. The research technical roadmap is shown in Fig 1. FE analysis was conducted on the uneven settlement stage, breaking columns stage and lifting columns stage of the building. FE modeling was applied to the correction areas of the building. Accurate material models were applied in FE model. Concrete damage plastic (CDP) model was employed for concrete material and elastic-plastic damage evolution model was used for steel material. Element sensitivity analysis ensured the reliability of the amount of mesh. The model was reasonably simplified, such as using the multi-layer shell element method to model the floor slab and simplifying welding joints with merging nodes method. This analysis included modal changes, overall displacement, stress disturbances, damage states, as well as internal forces and deformation results, providing a basis for understanding the potential effect of internal force release during the column truncation and lifting stages. During the processes of breaking columns and lifting columns, two breaking column methods and two lifting methods were employed. The effects of different methods on the structural stress field disturbances and the initial structural stability were investigated. A comparative analysis was conducted from the perspectives of displacement field and damage to highlight the differences in the impact on the structure between the two methods.

## 3. Uneven settlements of buildings

**Fig 2:**
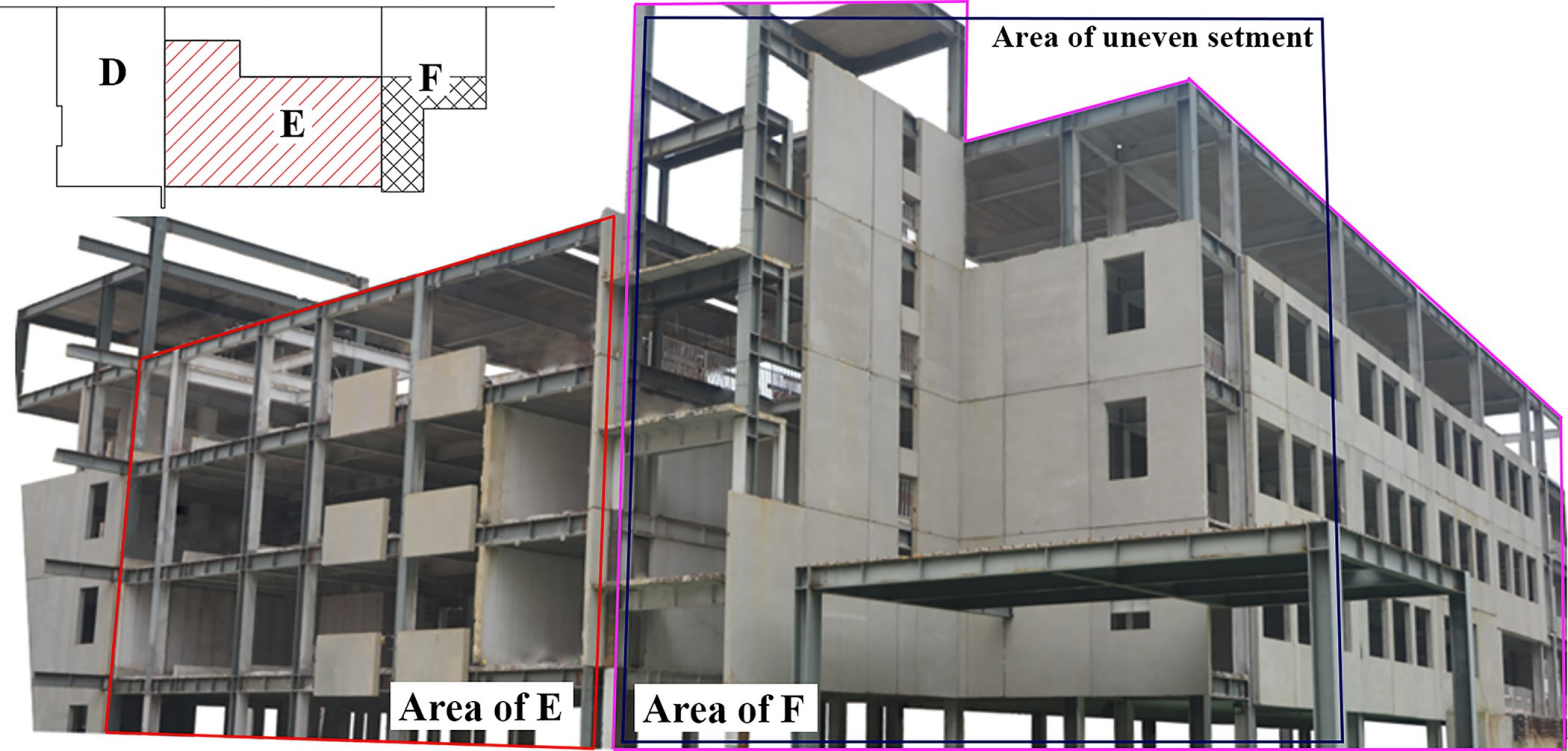
Realistic view of uneven settlement areas in buildings.

**Fig 3:**
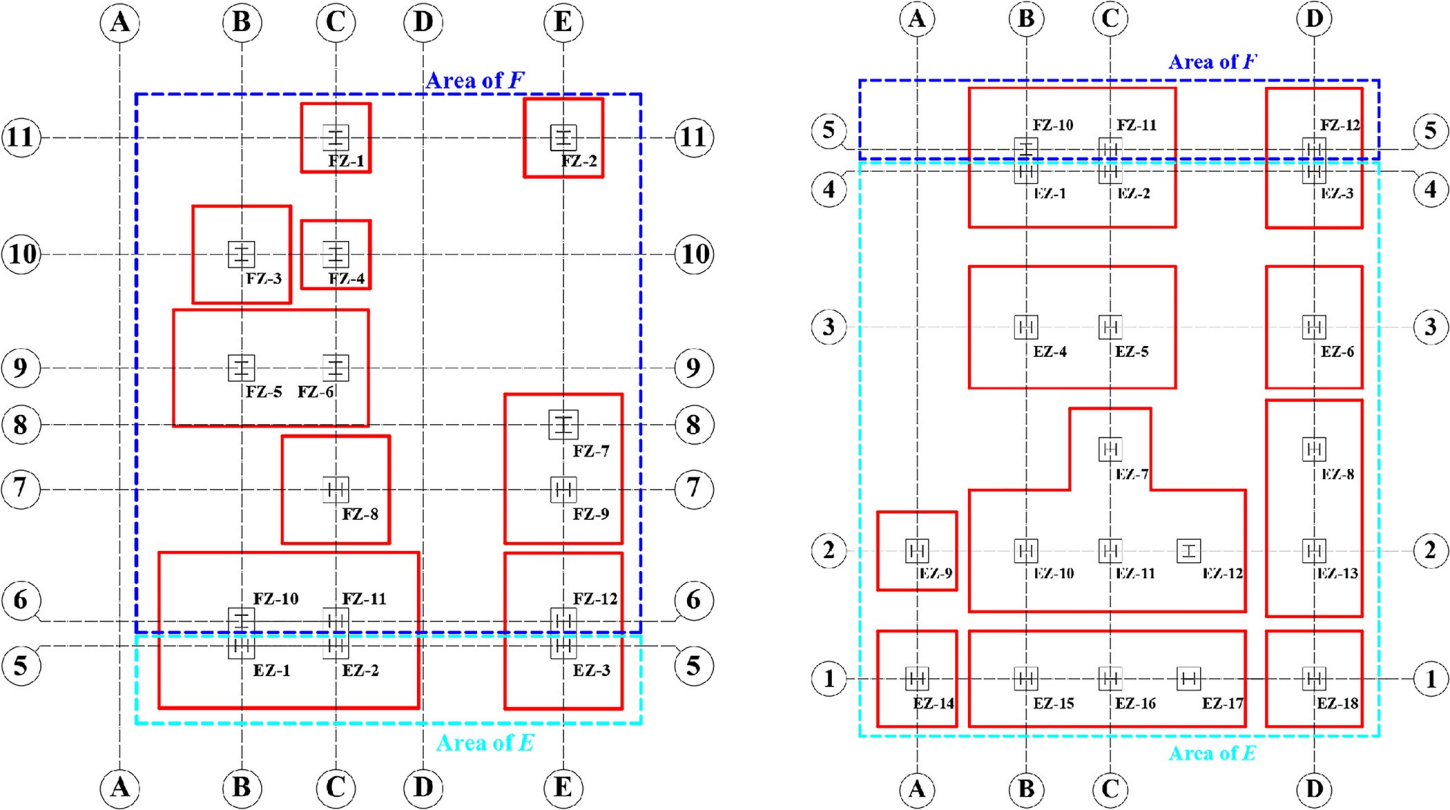
Axis distribution in uneven settlement areas (The direction up is in the north direction). (a) Area of E; (b) Area of F.

The main structure of a primary school in a certain city adopts a steel frame modular structure, which is an ongoing project, as shown in Fig 2. The overall structure has a maximum height of 23.5 m and a minimum height of 15.6 m. The building is a combination of 4-story and 6-story structures. Except for staggered floor slab design at the staircase locations, the floor slabs of other levels have consistent construction elevations. The maximum length and width of the building plane are 58.3 m and 43.04 m, respectively. The maximum span of the structure is 9 m, with a maximum number of spans being 6. Currently, uneven settlement has occurred in the *E* and *F* zones of the building. The *E* zone adopts independent foundations, while the eastern boundary between the *F* zone and the *E* zone has independent foundations, and the western part of the *F* zone has raft foundations. The *E* and *F* zones are depicted in Fig 3. The main focus of this study was to rectify buildings that have experienced uneven settlement. Therefore, the specific factors influencing uneven settlement in buildings were not considered in this study, such as environmental factors. The real scene of the uneven settlement area is shown in Fig 2, namely the areas of E and F in Fig 3.

**Fig 4:**
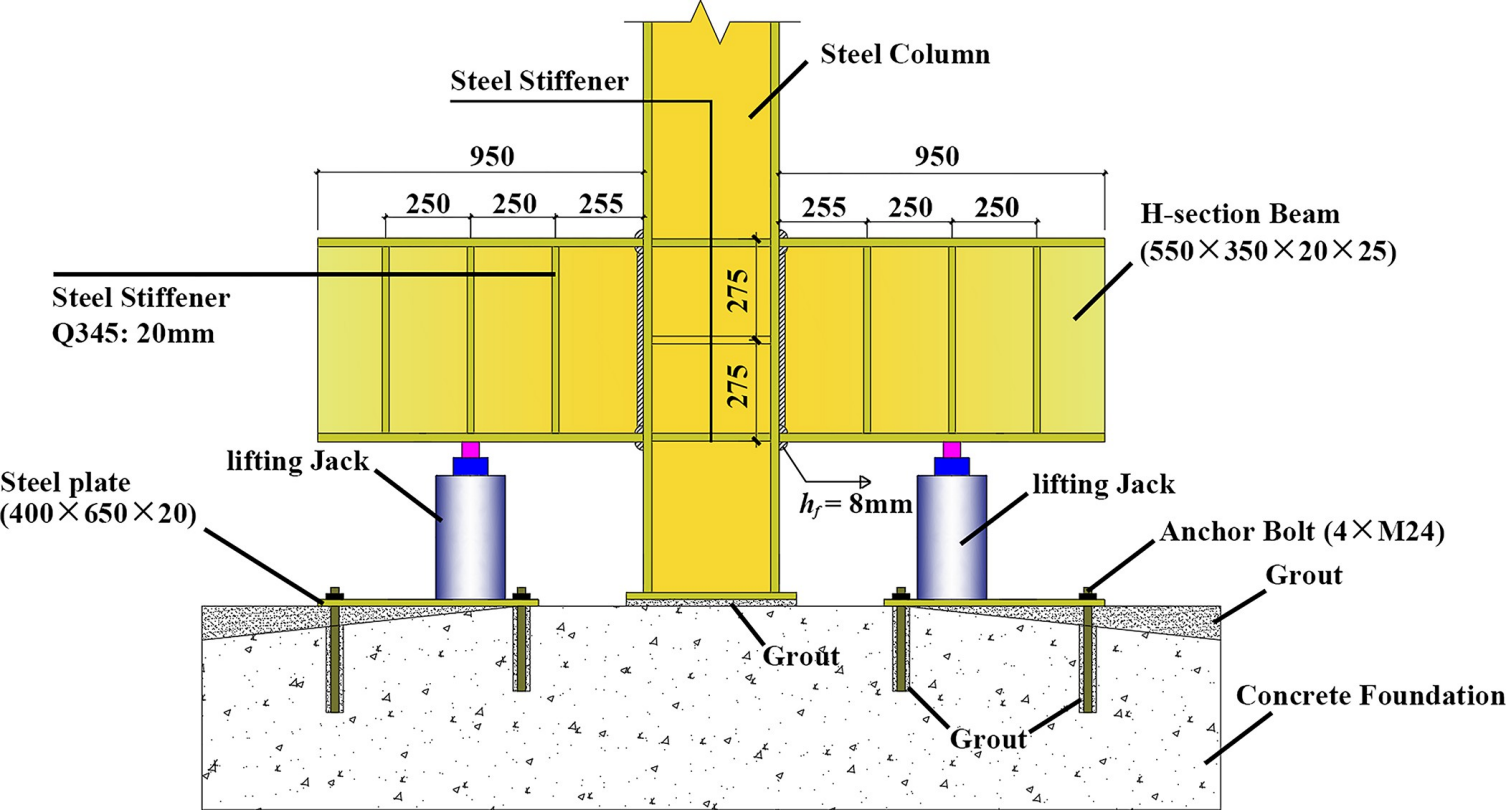
Schematic diagram for strengthening column bases.

**Fig 5:**
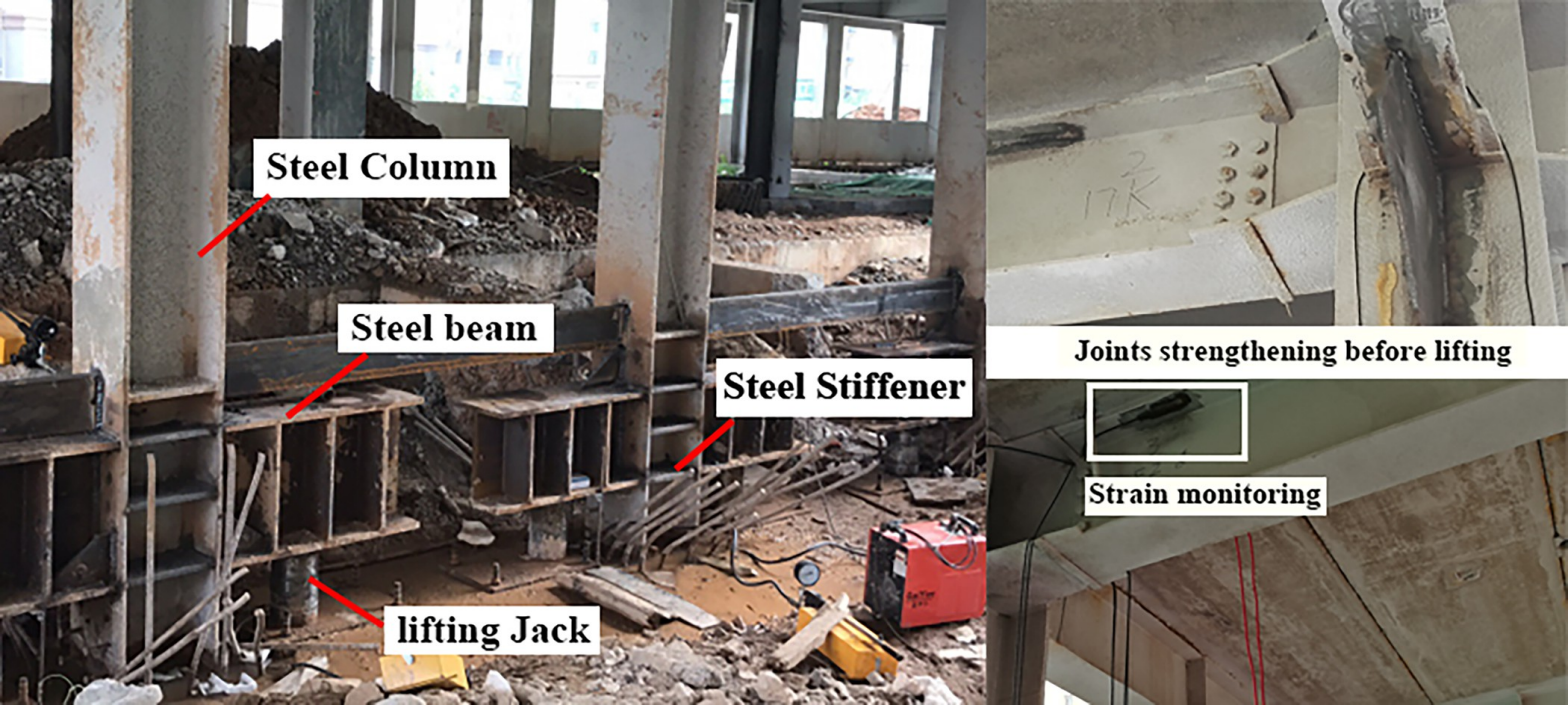
Realistic situation of strengthening column bases and steel joints.

According to the latest settlement observation data on site, the settlement amounts in the entire *E* zone and the eastern part of the *F* zone exceed the allowable values for foundation deformation of buildings specified in the Code for Design of Building Foundation (GB50007-2011) [26]. The plane of column distribution in the area where uneven settlement occurred was shown in Fig 3. The uneven settlement of every column can be found in Table 2. It can be observed that the settlement is greater on the eastern side compared to the western side, and the settlement on the southern side is slightly smaller than that on the northern side. The maximum settlement value is 247.61 mm, located at column E×8. The minimum settlement value is 26.31 mm, located at column A×1. The maximum settlement rate in the east-west direction of the building reaches 12‰, and in the north-south direction, it reaches 7.2‰, far exceeding the limit value 3‰ specified in the Code for Design of Building Foundation (GB50007-2011) [26]. Before the jacking process, it is necessary to cut off the column. After the column is severed, a specialized structure must be designed to ensure the jacking process, as illustrated in Fig 4. A short beam was configured on both sides of the column base. A jack was installed under each short beam. It should be noted that the column base was strengthened with steel stiffeners to avoid potential deformation, as shown in Fig 5. Some joints of the structure were strengthened with steel plates, and the strengthened positions depended on the results of numerical simulation. Meanwhile, strain gauges were installed to monitor local failures, as shown in Fig 5. During the process of breaking the column, it was recommended to connect the bottom of the column with a crossbeam to avoid significant deformation during stress release. After the columns were cut, the crossbeam needed to be removed.

**Table 2:** The uneven settlement of each column.

Area	Column ID	Cross-section	Settlement amount (mm)	Area	Column ID	Cross-section	Settlement amount (mm)
*C×11*	FZ-1	HW400×400×13×21	124.01	*B×4*	EZ-4	HW400×400×13×21	67.99
*E×11*	FZ-2	HW400×500×16×25	226.49	*C×4*	EZ-5	HW400×400×13×21	90.55
*B×10*	FZ-3	HW400×400×13×21	124.79	*E×4*	EZ-6	HW400×400×13×21	195.32
*C×10*	FZ-4	HW400×400×13×21	125.47	*C×3*	EZ-7	HW400×400×13×21	86.69
*B×9*	FZ-5	HW400×400×13×21	104.62	*E×3*	EZ-8	HW400×400×13×21	158.58
*C×9*	FZ-6	HW400×400×13×21	115.01	*A×2*	EZ-9	HW400×400×13×21	35.88
*E×8*	FZ-7	HW400×500×16×25	247.61	*B×2*	EZ-10	HW400×400×13×21	62.50
*C×7*	FZ-8	HW400×400×13×21	119.61	*C×2*	EZ-11	HW400×400×13×21	75.68
*E×7*	FZ-9	HW400×400×13×21	209.20	*D×2*	EZ-12	HW400×400×13×21	93.31
*B×6*	FZ-10	HW400×400×13×21	90.83	*E×2*	EZ-13	HW400×400×13×21	151.49
*C×6*	FZ-11	HW400×400×13×21	105.66	*A×1*	EZ-14	HW400×400×13×21	26.31
*E×6*	FZ-12	HW400×400×13×21	196.82	*B×1*	EZ-15	HW400×400×13×21	44.08
*B×5*	EZ-1	HW400×400×13×21	84.90	*C×1*	EZ-16	HW400×400×13×21	63.10
*C×5*	EZ-2	HW400×400×13×21	106.30	*D×1*	EZ-17	HW400×400×13×21	77.45
*E×5*	EZ-3	HW400×400×13×21	194.66	*E×1*	EZ-18	HW400×400×13×21	155.58

## 4. Finite element modeling

### 4.1. Modeling methods

**Fig 6:**
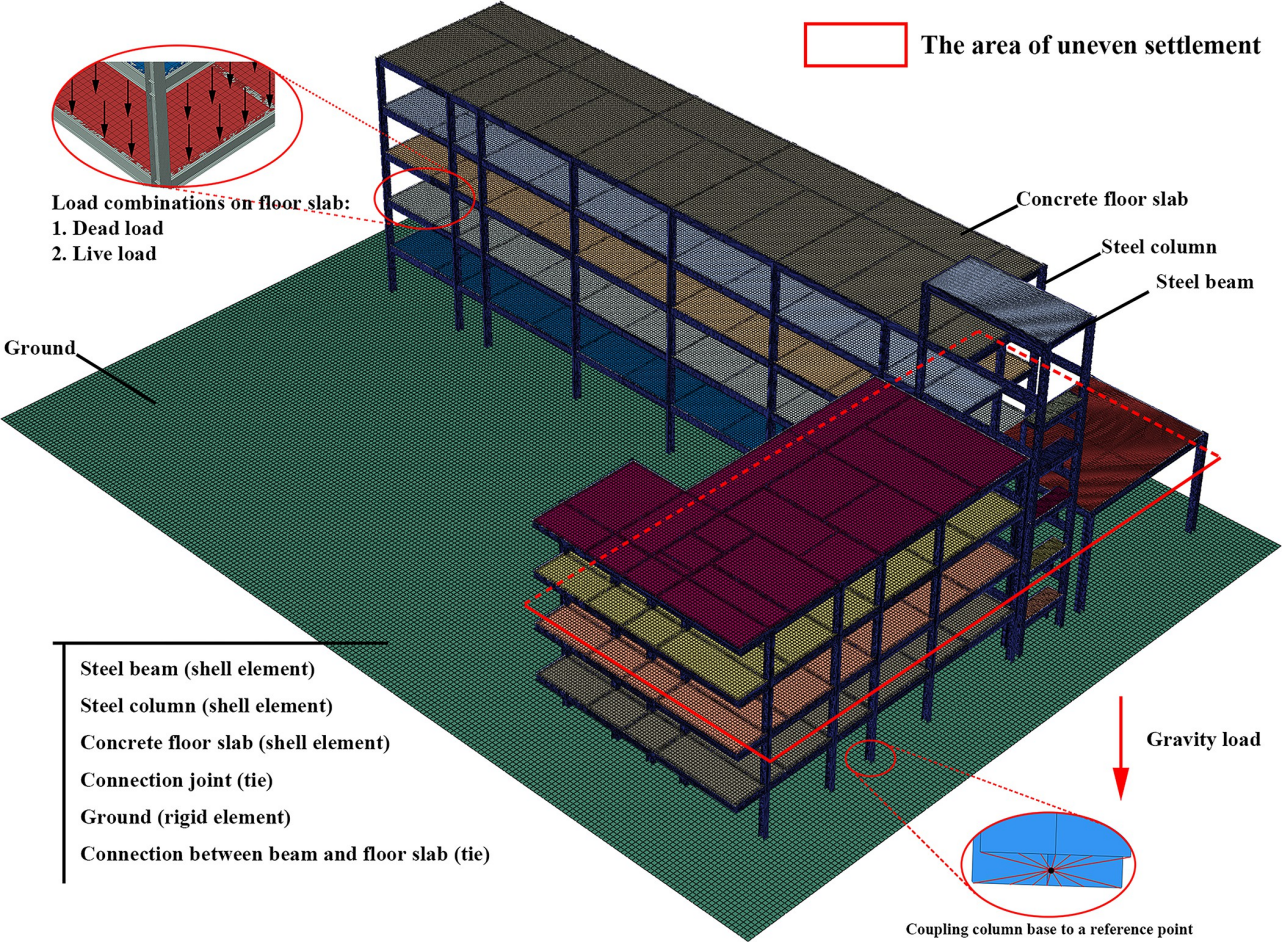
FE model of steel frame structure.

The large-scale general FE software ABAQUS was utilized for modeling in this study, as illustrated in Fig 6. The heights of the floors in area *E* were 3.73 m, 7.63 m, 11.53 m and 15.47 m, respectively. The heights of the floors in area *F* were 3.74 m, 7.76 m, 11.66 m and 15.56 m, respectively. The structure was a light steel-steel frame system, with the frame consisting of H-section steel beams and columns. The structural floor slab was composed of precast concrete slabs, and the exterior walls were also precast concrete. Shell elements were employed to simulate the steel beams, steel columns and concrete slabs, while beam elements were used to represent the strengthening. Shell elements were applied to model the beams and columns instead of beam elements and the mechanical performance of the components can be captured more accurately. The primary type of joint was welding connection. Therefore, the connection between the beams and columns can be simplified by merging nodes method. The thickness of the floor slab was 150 mm. The dimensions of the columns and beams have been listed to in Table 2. The primary dimensions of the columns were HW400×400×13×21, and the primary dimensions of the beams were H500×250×10×14. The material grade for beams and columns was Q345.

The primary interactions between components include the connection between rebar and concrete, as well as the interaction between the concrete slab and the steel frame. Tie connections and the embedded region method were used for simulating the contact and interaction. The tie method was employed between the floor slabs and the steel beams. Steel rebars were embedded in the concrete floor slabs to simulate the coupling effect. Uniform distributed loads were applied on the floor slabs, and line loads were applied on the steel beams to represent the gravity of the walls. According to the GB50007-2012 design code, the concrete floor slab was subjected to a uniform distributed load of 3.41 kN/m^2^. A linear load of 2.14 kN/m was applied on the steel beams to replace the mass of brick wall. Although contact between structural members was unlikely to occur, a potential automatic contact search was still set up. Normal and tangential contact properties were considered. Hard contact was defined in the normal contact direction. A friction coefficient of 0.2 was defined in the tangential contact direction. A structured meshing technique was adopted, and after mesh sensitivity analysis based on energy and structural response, the mesh size was set to 50 mm, with a total mesh count of 1,727,696. By controlling the degrees of freedom and displacements of the column base, breaking and lifting columns can be finished in FE models.

In this study, the different methods of breaking column and lifting columns were examined as key parameters. These methods included simultaneously breaking columns, chessboard style breaking columns, simultaneously lifting columns and lifting columns in multiple stages. The main focus of investigation was the difference of structural perturbations caused by four different methods.

The modeling approach needs to be adjusted according to different types of structures. The merging nodes method is not suitable for buildings with non-welded connections. For complex connection joint, the component method is recommended. In the case of prefabricated constructions, the using of the tie method between the floor slabs and steel beams will overestimate the restraining effect. Therefore, it is suggested to replace bolt connections with spring elements. In conclusion, the numerical simulation approaches should be determined based on the structural characteristics.

### 4.2. Mesh sensitivity analysis

**Fig 7:**
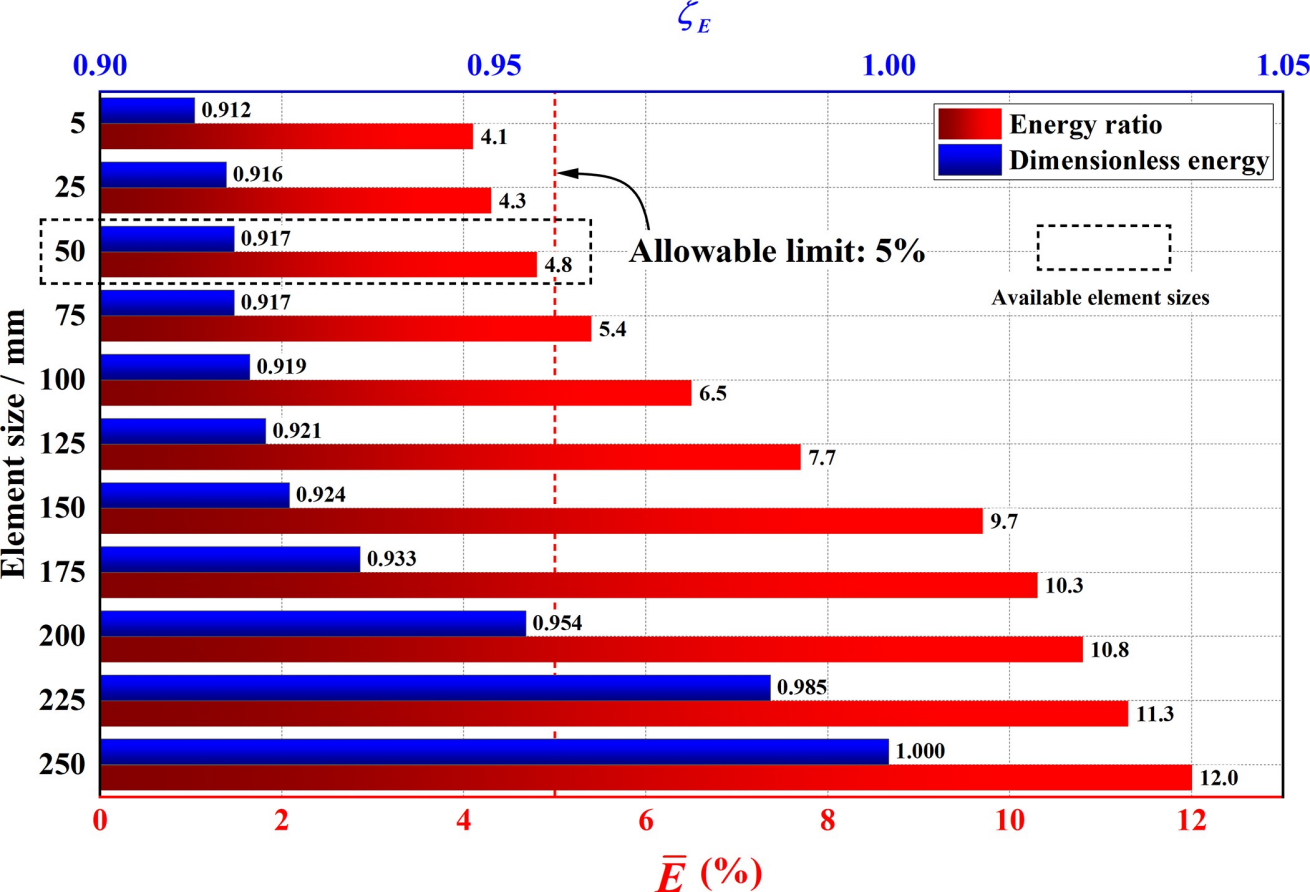
Results of element sensitivity analysis.

**Fig 8:**
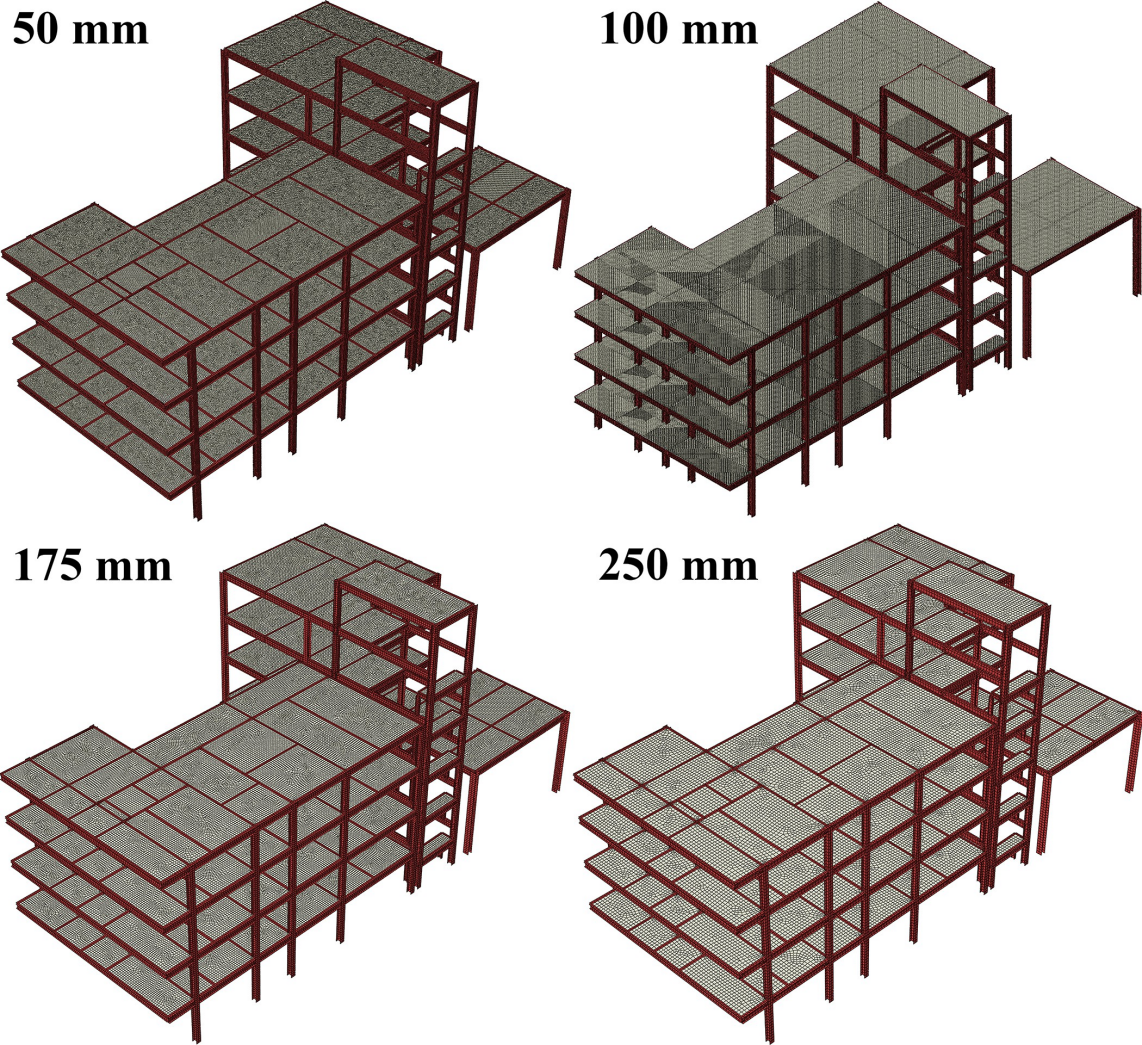
Mesh partitioning results.

In this section, an element sensitivity analysis is conducted to ensure that an appropriate mesh size is employed, and the numerical simulation results are accurate and reliable. The checking of element size ranges from 250 mm to 50 mm. The energy method is employed to assess the reliability of the results. Due to the use of reduced integration elements (S4R), the influence of artificial strain energy on the simulation results needs to be examined. Simultaneously, based on the principle that the structural total internal energy after uplifting should converge to a stable value, the sensitivity of the mesh is further investigated. *ζ*_*E*_ was defined as the normalized evaluation parameter based on internal energy of structural system with different element sizes. $\bar{E}$ was defined as the ratio of artificial strain energy to the total internal energy of the structural system. Two key parameters (*ζ*_*E*_ and $\bar{E}$) were calculated as shown in Eqs (1) and (2). The computed results are illustrated in Fig 7. The mesh partitioning results are shown in Fig 8. It can be observed that when the element size is less than 70 mm, $\bar{E}$ is less than 5%. Meanwhile, the impact of artificial strain energy can be neglected and the total internal energy of the overall structure tends to stabilize, indicating that the influence of element size can be disregarded. Therefore, an element size of 50 mm is deemed appropriate.

In summary, a structured meshing technique was adopted, and after mesh sensitivity analysis based on energy, the mesh size was set to 50 mm, with a total mesh count of 1,727,696.

$$\zeta_{E}=E_{ALLIE}^{\chi }/E_{ALLIE}^{\max}$$ (1)

$$\bar{E}=(E_{ALLAE}^{\chi }/E_{ALLIE}^{\chi })\times 100\%$$ (2)

where *χ* is the element size that checked in this section; *ALLAE* is the artificial strain energy; *ALLIE* is the internal energy; $E_{ALLAE}^{\chi }$ represents overall structural artificial strain energy when *χ* is employed; $E_{ALLIE}^{\chi }$ and $E_{ALLIE}^{\max}$ represent overall structural internal energy when element size is *χ* and the element size is 250 mm.

### 4.3. Constitutive models of materials

#### 4.3.1. Constitutive model of concrete

**Fig 9:**
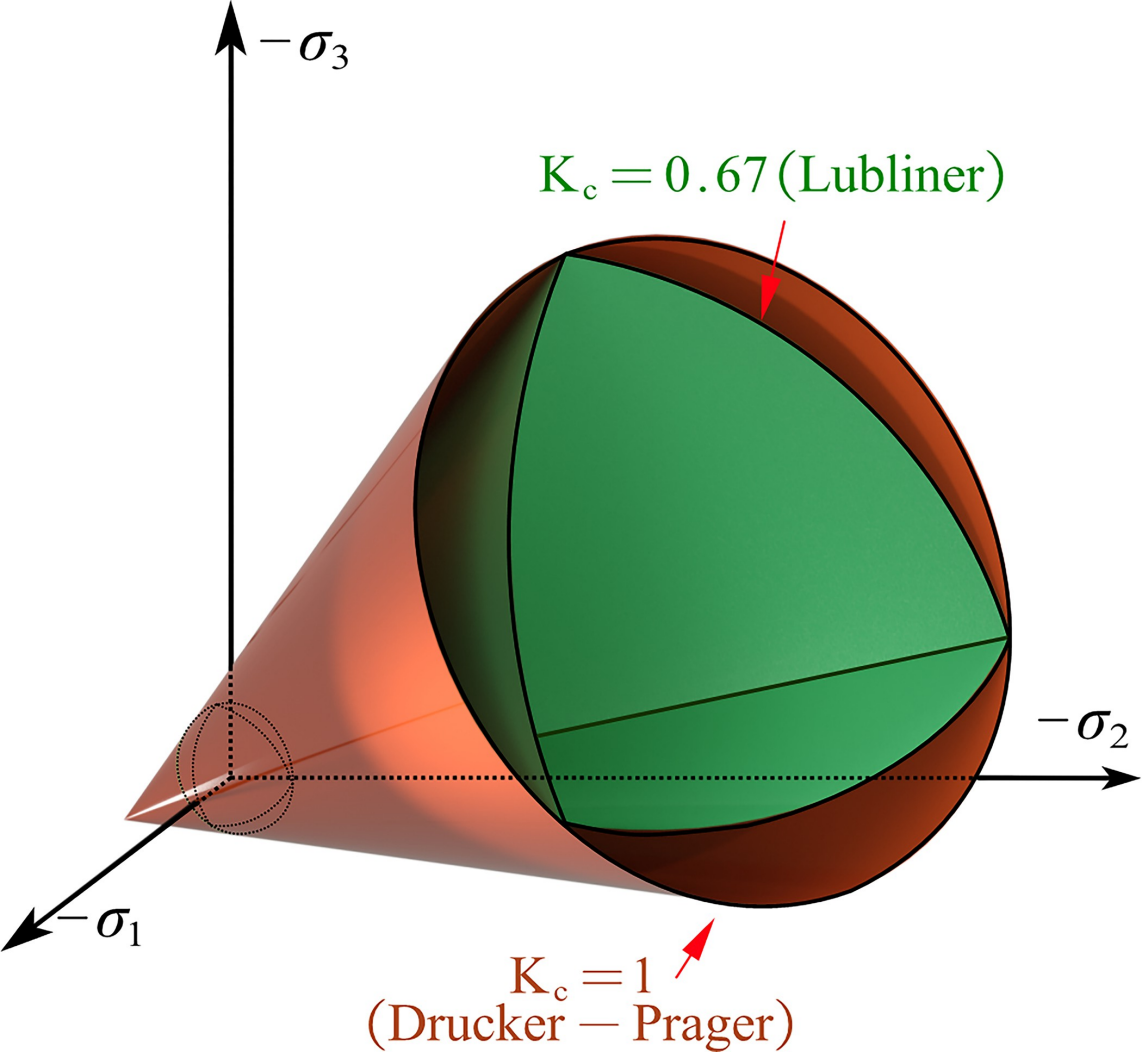
The yield surface in 3D stress space of CDP model.

In ABAQUS, the constitutive model of concrete can be implemented by selecting the material model and defining the corresponding material parameters. The Concrete Damage Plasticity (CDP) model for concrete was employed in this study, and the parameters and definitions of this model are detailed below. The CDP constitutive yield surface is illustrated in Fig 9. In the current simulation research on concrete structures with threaded steel or bolts, the CDP model demonstrates good applicability. The expression for material nonlinearity combines the concepts of isotropic elasticity in tension and compression with isotropic linear elasticity damage. The yield surface equation is derived from the modified Lubliner yield condition [27, 28].

$$F=\frac{1}{1-\alpha }[\sqrt{3J_{2}}+\alpha I_{1}+\beta \langle \sigma_{\max}\rangle -\langle -\sigma_{\max}\rangle ]-{\bar{\sigma }}_{c}({\bar{\varepsilon }}_{c}^{pl})=0$$ (3)

where $\alpha =\frac{(f_{\mathrm{b0}}/f_{\mathrm{c0}})-1}{2(f_{b0}/f_{c0})-1}$, $\beta =(1-\alpha )(f_{b0}/f_{c0})-(1+\alpha )$, $\gamma =\frac{3(1-K_{c})}{2K_{c}-1}$; *I*_1_ represents the first invariant of the stress tensor; *J*_2_ represents the second invariant of the deviatoric stress; *f*_*bo*_/*f*_*co*_ is the biaxial compressive strength of concrete relative to uniaxial compressive strength; ${\bar{\sigma }}_{c}({\bar{\varepsilon }}_{c}^{pl})$ is the effective viscous stress; *K*_c_ is the shape parameter of the yield surface on the π-plane. Specifically, when the maximum principal stress is negative, it is the ratio of the second stress invariant *q*(TM) on the tension *p-q* plane to that on the compression plane *q*(CM). It must satisfy the condition 0.5<*K*_c_≤1.0. Based on extensive experimental data [29], fitted the values of *K*_c_ and *f*_*bo*_/*f*_*co*_, providing the following empirical formulas:

$$K_{c}=0.71f_{c}^{-0.025}$$ (4)

$$f_{bo}/f_{co}=1.57f_{c}^{-0.09}$$ (5)

The CDP model adopts a non-associated flow rule, with the flow potential function *G* being the Drucker-Prager hyperbolic function:

$$G=\sqrt{{\left(\in \sigma_{t0}\tan\psi \right)}^{2}+{\bar{q}}^{2}}-\bar{p}\tan\psi$$ (6)

where *p* and *q* represent the static hydrostatic pressure and von Mises equivalent stress in the effective stress space, respectively; *σ*_*to*_ is the uniaxial tensile stress at failure, obtained from the provided tensile hardening data. *ψ* is the dilation angle on the *p*-*q* plane; *∈* is the eccentricity of the plastic potential function for concrete material, defining the rate at which the function approximates the asymptotic line. When the eccentricity approaches 0, the plastic flow potential tends towards a straight line. The default value of eccentricity in ABAQUS is 0.1, implying that under allowable confinement, the dilation angle of concrete hardly changes. Severe convergence issues arise when the eccentricity is less than 0.1. Therefore, eccentricity is not further adjusted here and is set to 0.1.


$$\psi =\tan^{-1}[\frac{6(0.362-8\times 10^{-6}f_{c}^{2}-2\times 10^{-4}f_{c})}{\frac{3E_{c}\varepsilon_{cp}}{f_{c}}+2(0.362-8\times 10^{-6}f_{c}^{2}-2\times 10^{-4}f_{c})-3}]$$ (7)

The compressive peak strain of concrete is calculated by Eq (8).


$$\varepsilon_{cp}=3.78\frac{f_{c}^{0.75}}{E_{c}}{\left(\frac{\rho_{c}}{2320}\right)}^{0.3}$$ (8)

Plasticity parameters of CDP model need definitions for the yield surface parameter (*K*_c_), the ratio of uniaxial to biaxial compressive strength (*f*_*bo*_/*f*_*co*_), dilation angle (*ψ*), eccentricity (*∈*), and viscosity parameter (*μ*). The concrete material density is set to 2400 kg/m^3^, and the Poisson’s ratio is 0.2. The elastic modulus can be determined using the Eq (9). In this study, the CDP model parameters are presented in Table 3. The parameters of concrete CDP model referred to the published research as the same materials were applied [30].


$$E_{c}=(3320\sqrt{f_{c}}+6900){\left(\frac{\rho_{c}}{2320}\right)}^{1.5}\;(MPa)$$ (9)

**Table 3:** The material parameters of concrete CDP model.

Expansion angle / *ψ*	*f*_bo_ / *f*_co_	Eccentricity / ∈	*K*_c_	Viscosity parameters / *μ*
37	1.07	0.1	0.67	0.0005

**Fig 10:**
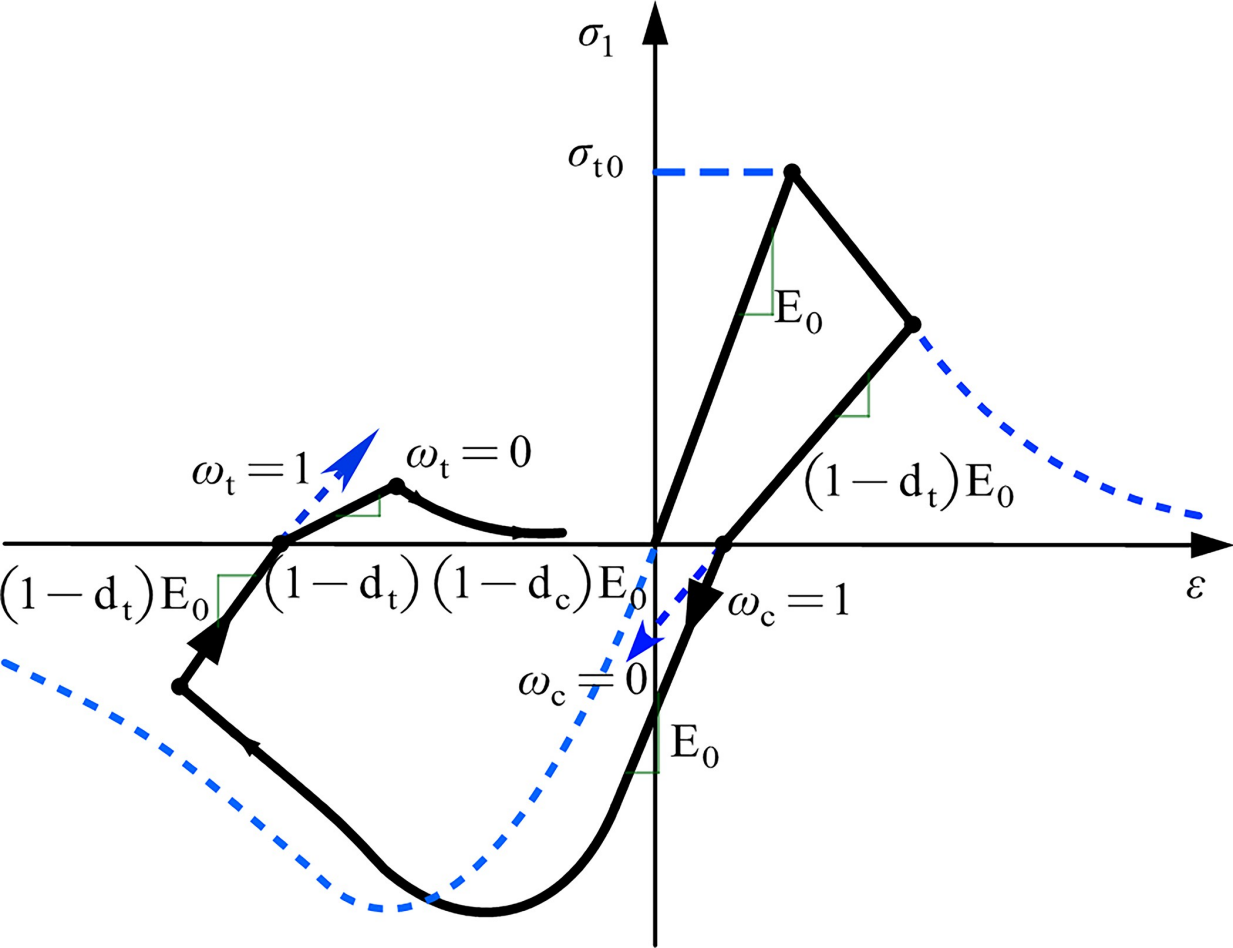
Uniaxial load cycle curves of concrete material model [33].

The CDP model, based on material response characteristics, reasonably simplifies the uniaxial loading behavior. During the softening stage, when the material unloads, the damage behavior is characterized by reducing the elastic modulus. This is achieved through the damage factors (*d*_c_ and *d*_t_) to represent the degree of stiffness degradation [31]. *w*_c_ and *w*_t_ are used to characterize the stiffness recovery parameters, as shown in Fig 10.

#### 4.3.2. Constitutive model of steel

**Fig 11:**
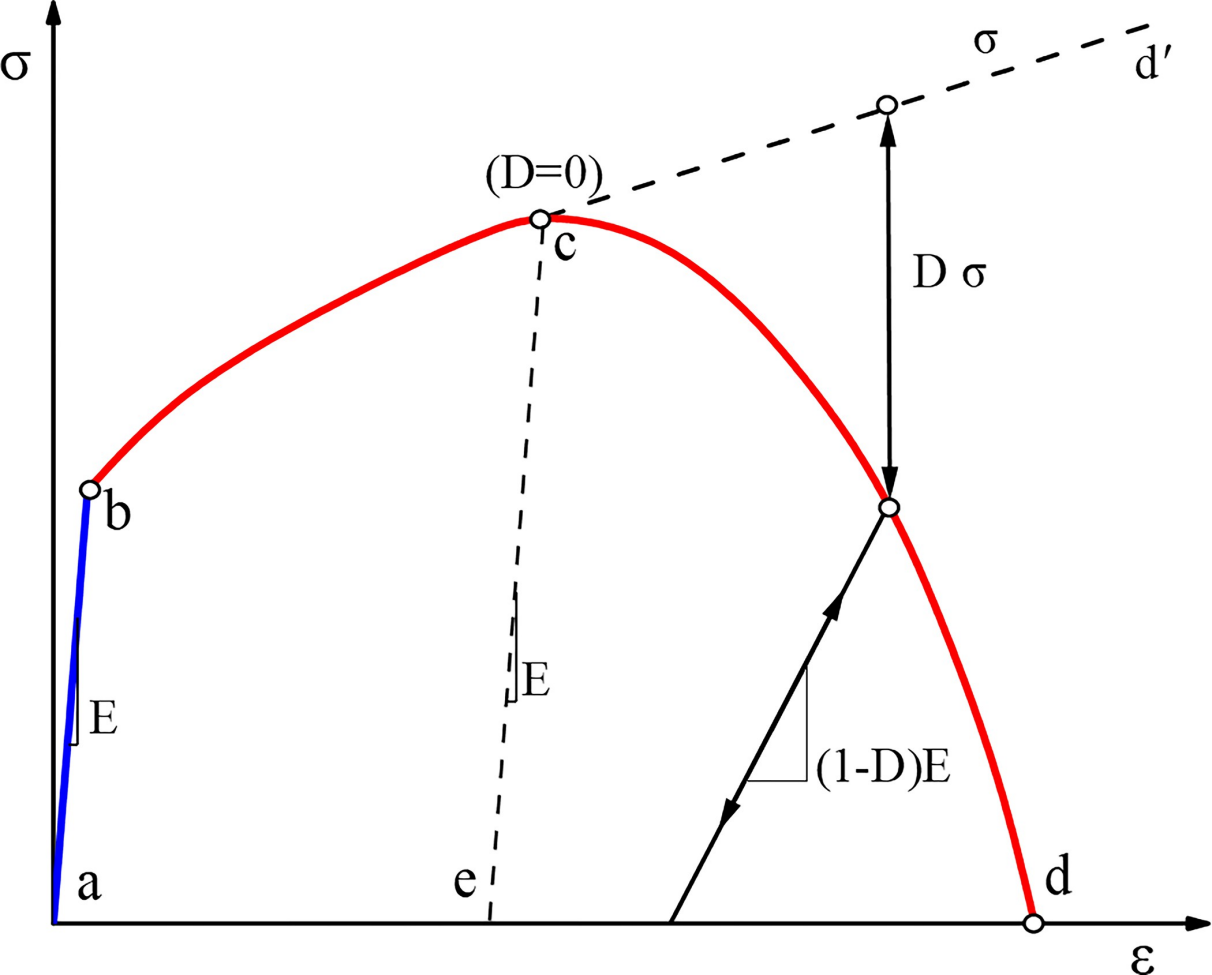
Plasticity and damage evolution curves of steel material model [34].

For steel material, a refined constitutive model is employed, encompassing elastic-plastic stages, damage evolution and failure stages. Stress hardening and softening effects are also considered, as shown in Fig 11. The engineering stress-strain curve can be obtained from uniaxial tensile tests on the material. True stress-strain data usable in ABAQUS can be derived based on Eq (10).


$$\{\begin{array}{l} \sigma_{true}=\sigma_{eng}(1+\varepsilon_{eng})\\ \varepsilon_{true}=\ln(1+\varepsilon_{eng}) \end{array}$$ (10)

Sections *a*~*b* in Fig 11 represent the elastic stage of the material, while the *b*~*d* corresponds to the plastic strengthening phase without considering damage evolution. When the damage evolution is considered, point *c* is the initiation of damage, marking the onset of damage evolution and stiffness reduction. The overall damage variable *D* and individual damage variable *d*_i_ are defined. In the presence of multiple damage evolution criteria, an intermediate damage variable *d*_mult_ is calculated according to Eqs (11) and (12) to compute the overall damage variable *D* [32]. When Eq (13) is satisfied, the material stiffness is considered to have been completely lost [32].


$$d_{mult}=1-\prod_{i\in N_{mult}}\left(1-d_{i}\right)$$ (11)

$$D=\max\{d_{mult},\underset{j\in N_{mult}}{\max}(d_{j})\}$$ (12)

D≥1 (13)

The initiation of damage adopted a stress triaxiality-dependent computational model, considering both ductile and shear damage, as shown in Eqs (14) and (15). After the onset of damage, the stiffness degradation rate (referred to as "*D*" in Fig 11) was used to characterize the decrease in material stiffness. SDEG was defined as the overall damage variable, i.e., parameter *D* in Eqs (12) and (13). When SDEG > 0, damage begins to occur in the material, and when SDEG = 1, the material is considered fully damaged. The process of damage evolution employs an energy-based approach, with the calculation of fracture energy (*G*_f_) given by Eq (16).


$$\frac{{\bar{\varepsilon }}_{D}^{p}}{\varepsilon_{u}}=\{\begin{array}{l} 1.13\\ 0.04+0.86\exp(-0.7\eta )\\ 0.12 \end{array}\;\begin{array}{c} \eta \leq -1/3\\ -1/3\leq \eta \leq 10/3\\ 10/3\leq \eta \end{array}$$ (14)

$$\frac{{\bar{\varepsilon }}_{S}^{p}}{\varepsilon_{u}}=\{\begin{array}{l} 0.43\\ 0.38+0.40\exp[6.69(\eta -2)]\\ 0.78 \end{array}\;\begin{array}{c} \eta \leq 5/3\\ 5/3\leq \eta \leq 2\\ 2\leq \eta \end{array}$$ (15)

$$G_{f}=\int_{{\bar{\varepsilon }}_{o}^{pl}}^{{\bar{\varepsilon }}_{f}^{pl}}L\sigma_{y}d{\bar{\varepsilon }}^{pl}$$ (16)

### 4.4. Model validation and reliability analysis

In this section, the reliability of the FE models was checked based on the data measured in engineering practice compared with the numerical simulation results. A comparison of strain gauges measurements and FE results was provided in Table 4. The results shown that the error between the measured values from strain gauges and the numerical simulation results was less than 21%. The overall trend was that the numerical simulation results were slightly larger than the measured values. However, this error was acceptable because the numerical simulation results were on the conservative side, i.e., on the safe side for the engineering scheme design.

**Table 4:** Comparison of measured and numerically simulated results for strain gauges.

Position of strain gauges	*ε*_*Measure*_ (10^−6^)	*ε*_*FE*_ (10^−6^)	Error (%)	Position of strain gauges	*ε*_*Measure*_ (10^−6^)	*ε*_*FE*_ (10^−6^)	Error (%)
*C×11*	1761	1955	11	*B×5*	1970	2364	20
*B×10*	1196	1351	13	*B×4*	1669	1853	11
*B×9*	203	223	10	*C×3*	502	542	8
*E×8*	1318	1489	13	*E×3*	680	775	14
*C×7*	1338	1539	15	*A×2*	1227	1423	16
*B×6*	1845	1974	7	*C×1*	946	1078	14

Note: *ε*_*Measure*_ represents the strain from the measurement of strain gauges; *ε*_*FE*_ represents the strain from FE calculation.

## 5. The impact of uneven settlement on structures

### 5.1. Modal and frequency analysis

The modal changes before and after structural settlement can be used to characterize the variations in the inherent properties of the structure post-deformation. This can be employed to assess the stability of the structure in the absence of external forces and its potential dynamic response characteristics. The modal changes between the initial and deformed states of the structure can be computed using the Frequency analysis step in the FE analysis software ABAQUS. When the structure undergoes deformation, its initial state changes, thereby influencing the modal characteristics of the structure.

**Fig 12:**
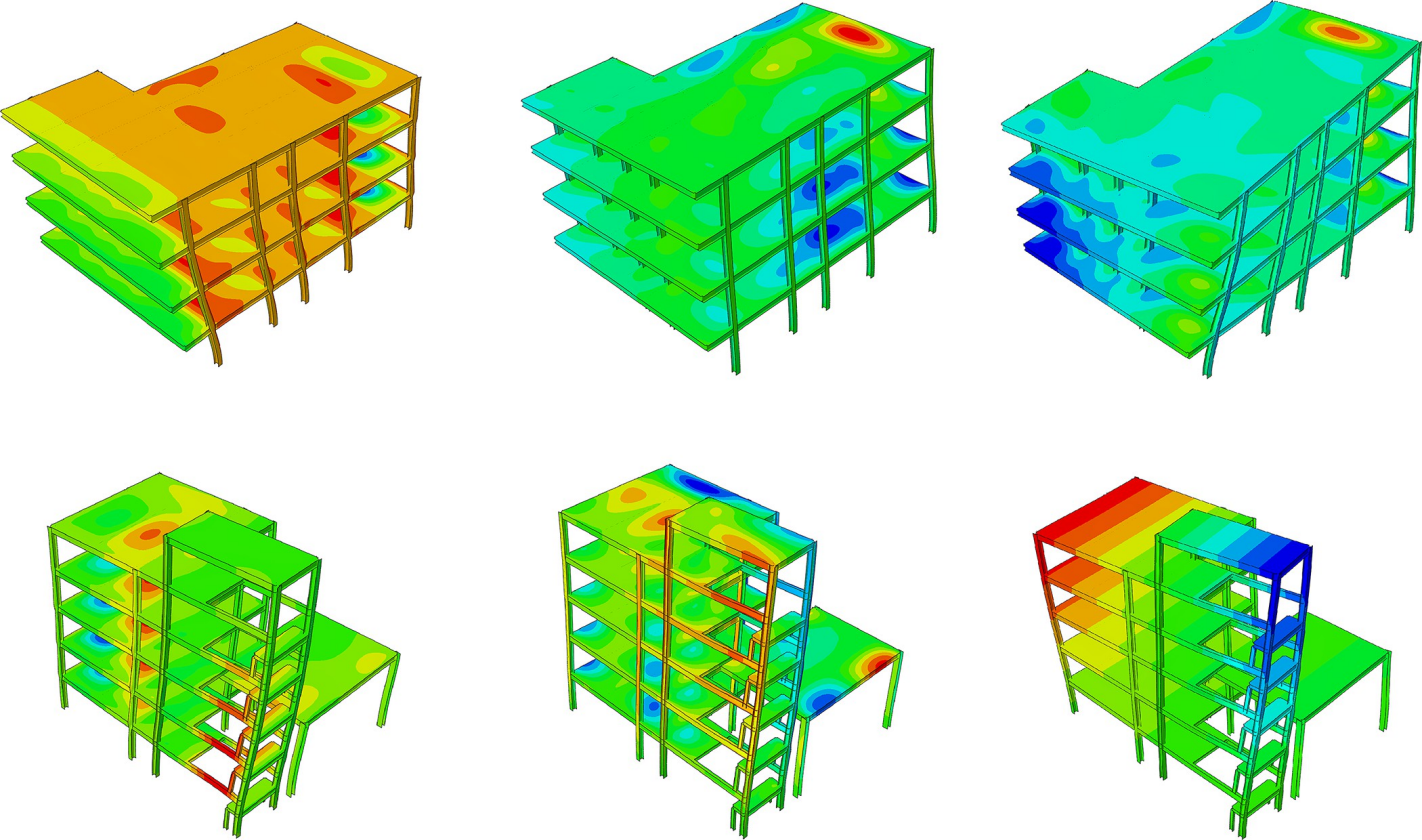
The first three modes of areas E and F. (a) first mode of area E; (b) second mode of area E; (c) third mode of area E; (e) first mode of area F; (f) second mode of area F; (g) third mode of area F.

**Fig 13:**
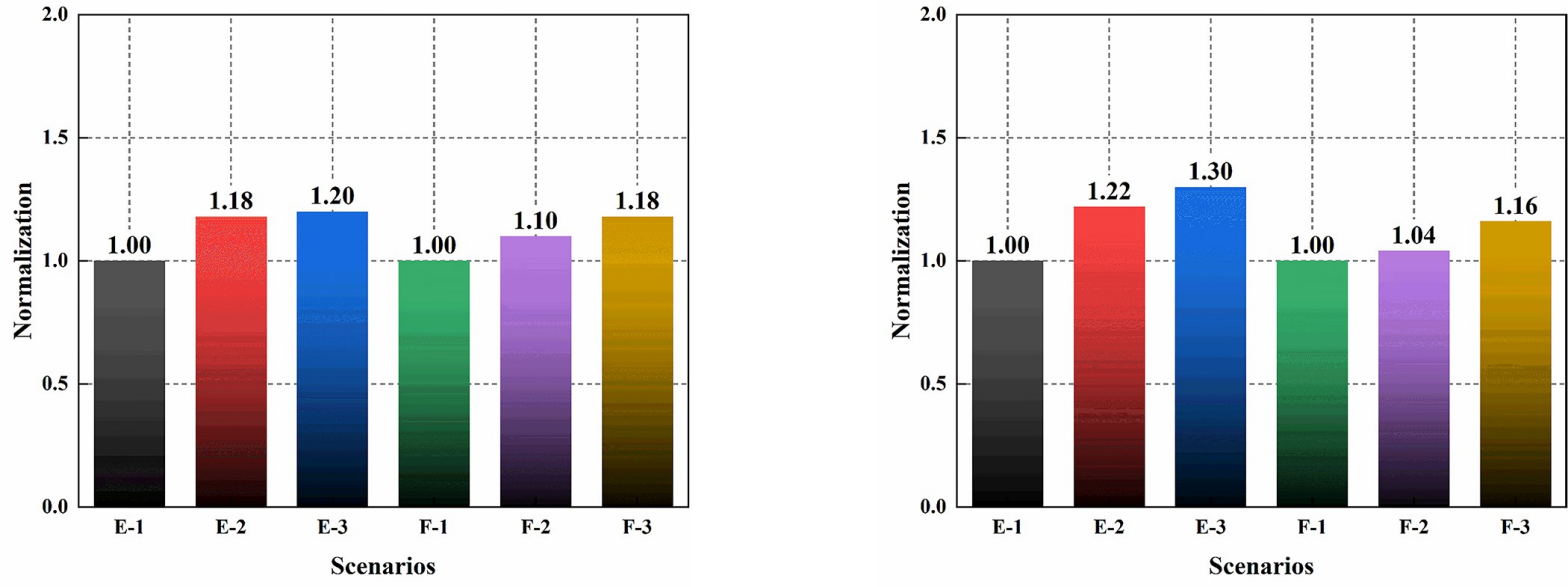
Structural frequency analysis and comparison. (a) Structural frequency before settlement; (b) Structural frequency after settlement.

The first three modes of regions *E* and *F* under static loading is illustrated in Fig 12. It can be observed that the first three modes in region *E* primarily represent the overall deformation of the structure. The first two modes correspond to translations in two directions, and the third mode involves the overall torsion of the structure. There is no significant difference in the modal shapes before and after settlement. Fig 13 presents the normalized frequency growth of the modes before and after settlement. The maximum frequency growth rate of the second mode compared to the first mode was 22%, and the maximum frequency growth rate of the third mode compared to the first mode was 30%. A smaller growth rate meant more similar structural features in the direction being examined. Smaller discreteness was advantageous for the structural safety after being perturbation. Comparing the frequencies and eigenvalues of the first three modes before and after settlement, as shown in Table 5, reveals that, before settlement, for region E, compared to the eigenvalue of the first mode, the eigenvalues of the second and third modes are approximately 38% and 43% higher, with frequencies about 17% and 19% higher, respectively. For region F, compared to the first mode’s eigenvalue, the second and third mode eigenvalues are approximately 21% and 39% higher, with frequencies about 10% and 17% higher, respectively. After uneven settlement, the first three modes in region E decrease by 2.03%, 6.23% and 2.71%, respectively. After uneven settlement, the first three modes in region F decrease by 2.17%, 7.41% and 1.15%, respectively.

**Table 5:** Modal analysis before structural settlement.

Area ID	First mode		Second mode		Third mode	
	Eigenvalue	Frequency	Eigenvalue	Frequency	Eigenvalue	Frequency
*BS-E*	238.62	2.46	329.83	2.89	342.53	2.95
*AS-E*	228.72	2.41	266.67	2.71	301.50	2.87
Error	4.15	2.03	19.15	6.23	11.98	2.71
*BS-F*	192.62	2.21	233.24	2.43	267.54	2.60
*AS-F*	171.68	2.16	220.86	2.25	258.07	2.57
Error	10.87	2.17	5.31	7.41	3.54	1.15

Note: “*BS*” and “*AS*” represent “before settlement” and “after settlement”; “*E*” and “*F*” represent “area *E*” and “area *F*”.

### 5.2. Stress field and damage analysis

**Fig 14:**
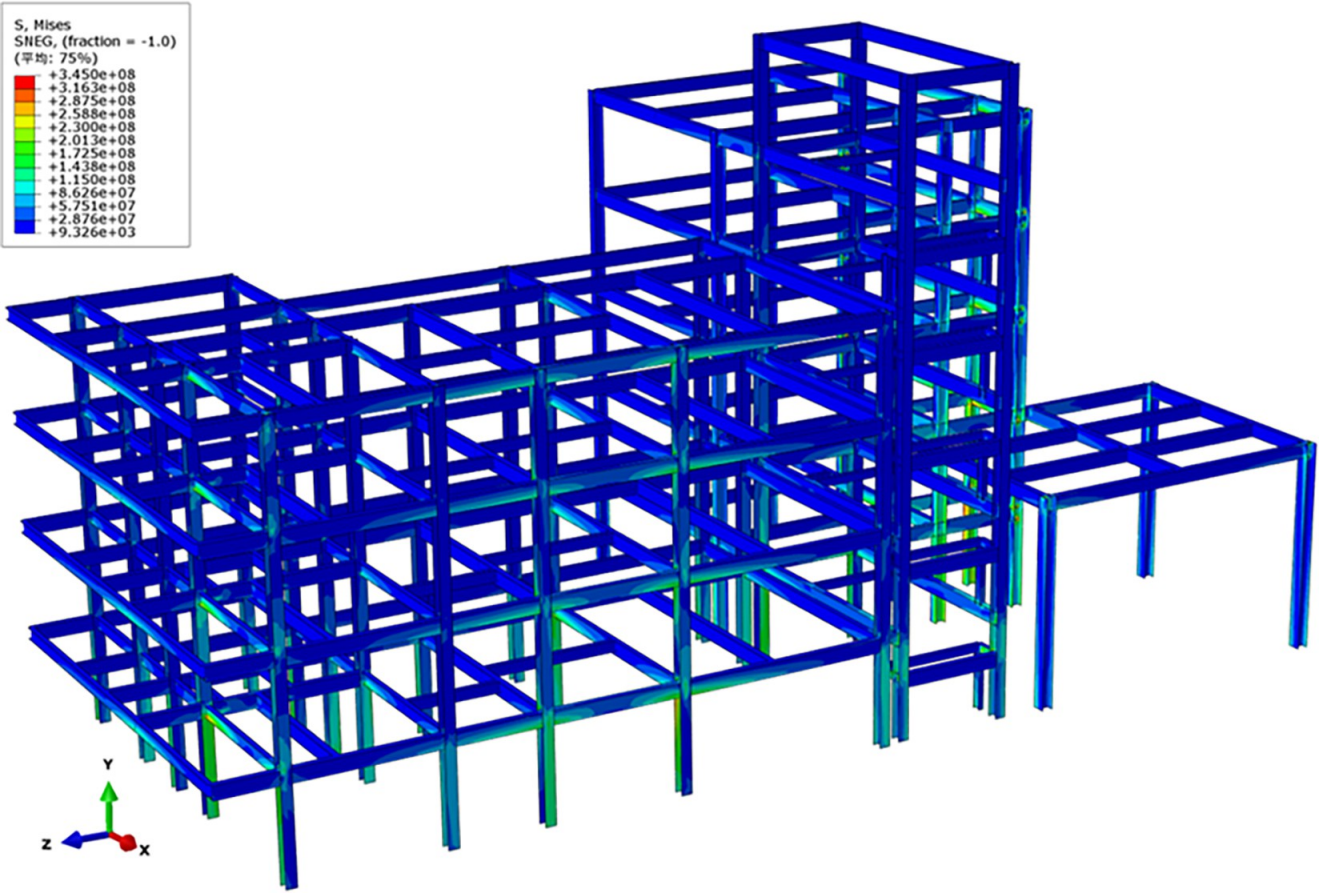
Von Mises stress field of steel frame structures (unit: Pa).

**Fig 15:**
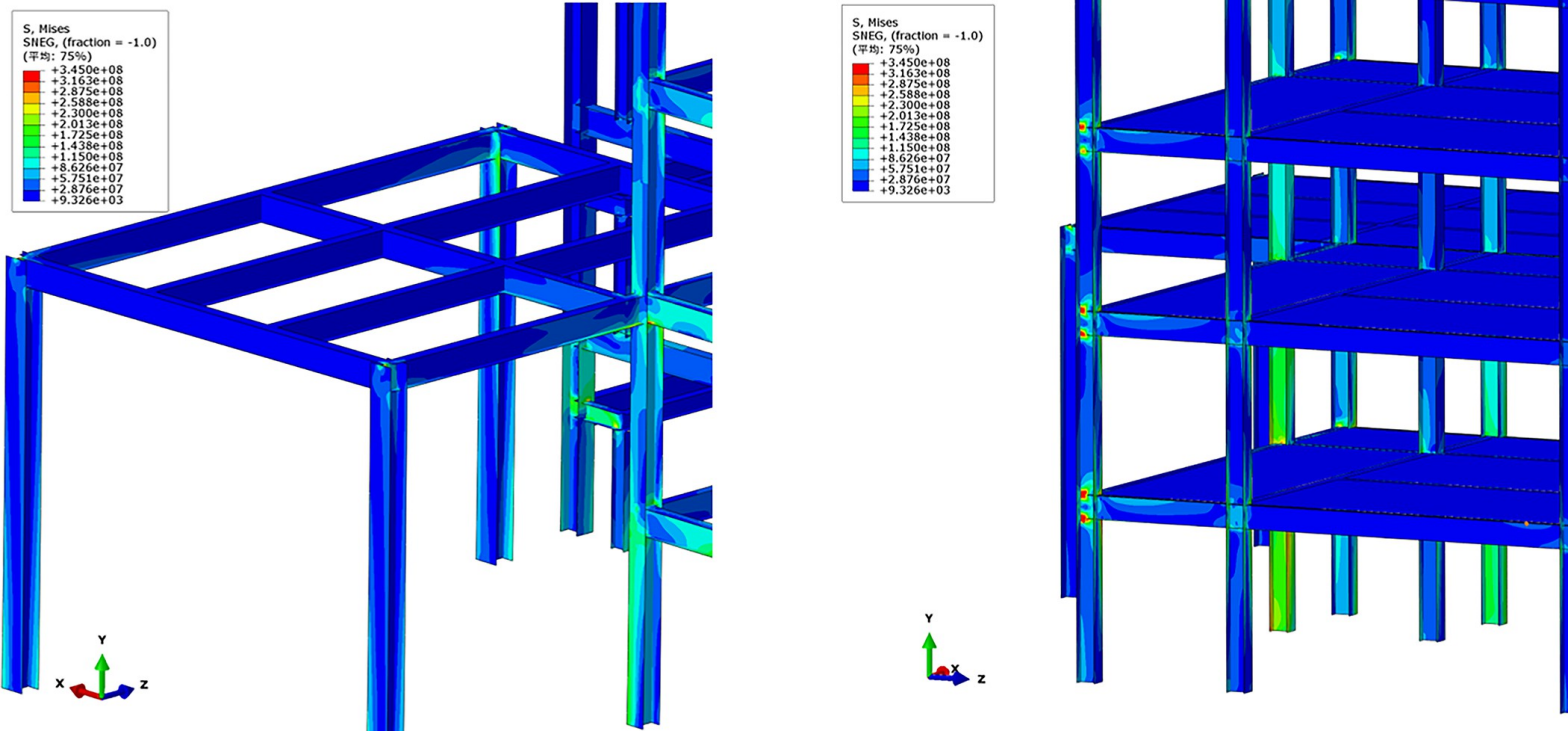
Stress concentration and damage in area F (unit: Pa).

Based on the refined FE model established in the previous section, FE structural stress field calculations and inspections of stress concentrations were conducted to directly assess the impact of uneven settlement on the structure. Fig 14 depicts the von Mises stress field of the steel frame, used to analyze stress disturbances within the steel frame after settlement. Fig 15 illustrates the local stress concentration distribution of the steel frame structure. It can be observed that, for the building structure in Zone *E*, stress concentrations resulting from uneven settlement are mainly concentrated in areas with significant settlement, such as EZ-18, EZ-13, EZ-8, EZ-6, EZ-3. The connection nodes between the column group and frame beams in these areas experience severe stress concentrations, with some regions of the material entering the plastic stage. Regarding the building structure in Zone *F*, stress concentrations caused by uneven settlement are primarily focused on areas with substantial settlement, such as FZ-9, FZ-7, FZ-2, FZ-1, FZ-3. Particularly, the connection joints between the outer columns and staircase beams exhibit pronounced stress concentration phenomena, with the material in the stress concentration region exceeding the yield stress.

## 6. Calculation and analysis of building inclination rectification process

**Fig 16:**
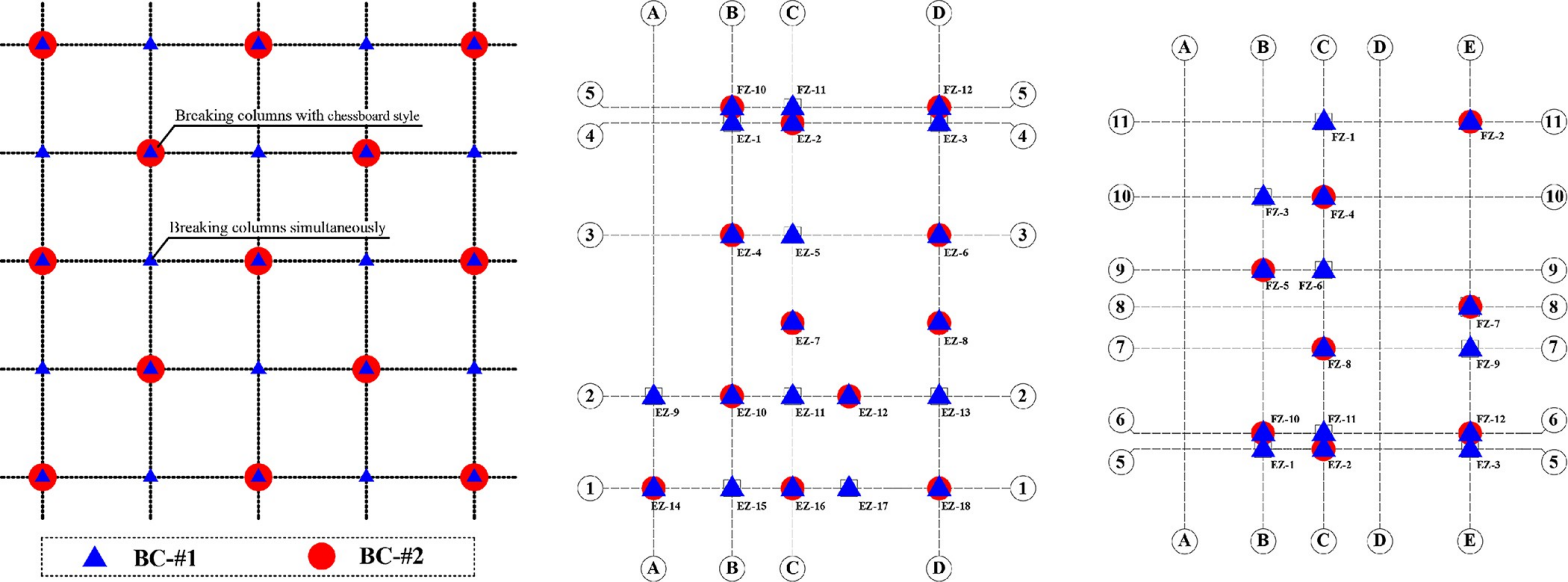
Different breaking columns methods. (a) Schematic of different methods; (b) Realistic situations of breaking columns in areas E and F.

**Fig 17:**
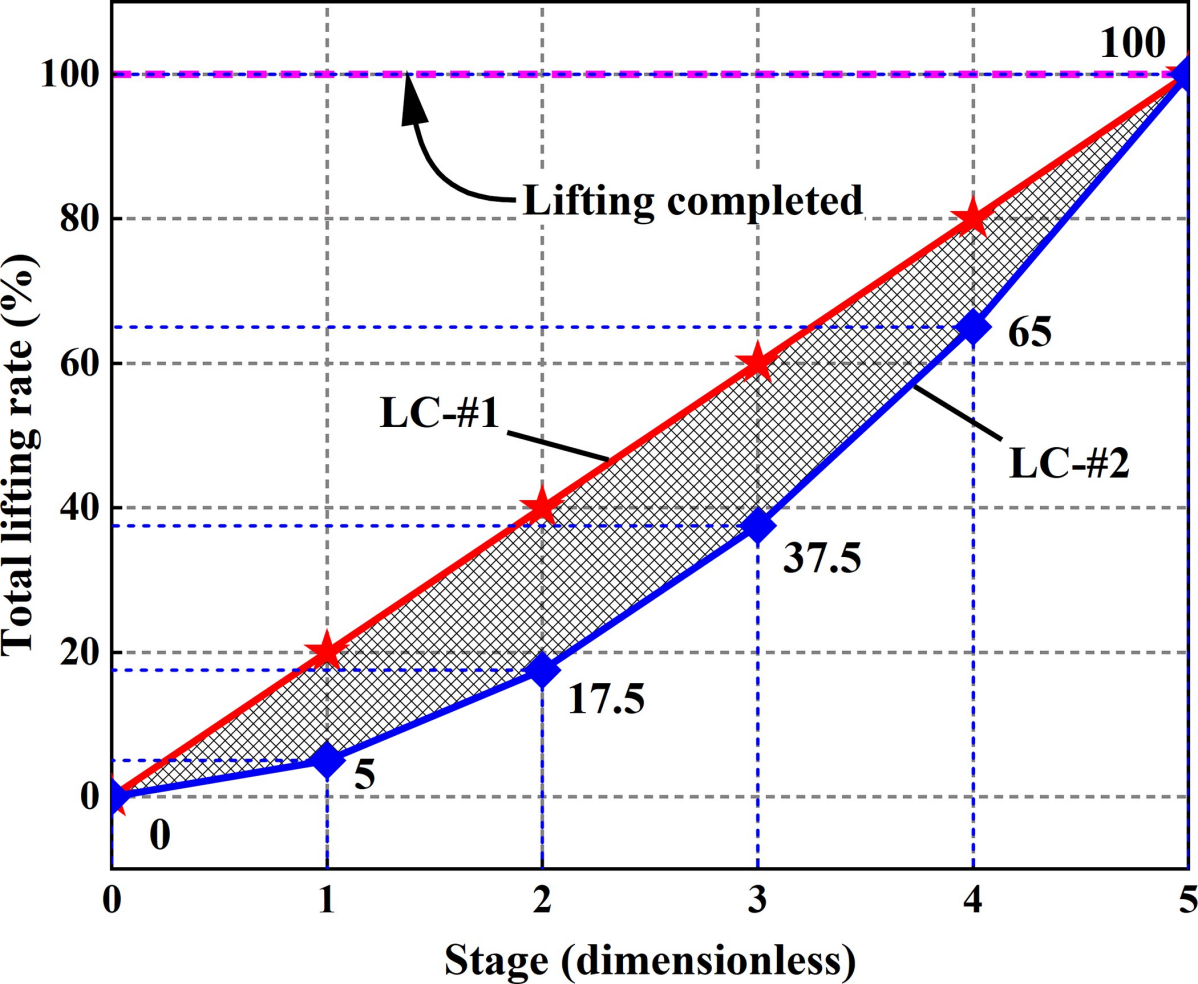
Different lifting columns methods and load path.

In this section, a FE method will be employed to analyze the perturbation of structural stress during the column removal and lifting process. Two column removal methods, labeled as BC-#1 and BC-#2, and two lifting schemes, denoted as LC-#1 and LC-#2, are considered, as shown in Table 6. The specific schematic representations of the four methods are shown in the Figs 16 and 17. Fig 17 shows the path of the percentage displacement of the column base during the lifting process. The process of lifting was divided into a total of 5 stages. The applied displacement of each stage can be calculated by multiplying the total of lifting displacement of each column by the percentage of each stage. It should be noted that multiple restart FE models were required for LC-#2 to achieve the multi-level lifting. However, only required one FE model was required for LC-#1. The effect of different column removal methods and lifting schemes on structural perturbations are examined, thereby validating the reliability of the adopted restoring techniques. Due to the fact that structural damage arises from secondary structural disturbances caused during the process of breaking columns. BCs methods significantly affect the stress release within the structure, while in uplifting methods only influence the final deformation model of the structure. Therefore, the disturbances to the structure under different BCs methods will be examined and discussed in Section 6.1. The effects of different LCs methods on structural disturbances will be discussed in Section 6.2.

**Table 6:** Methods of broken and lifting columns.

The method of breaking columns (BC)		The method of lifting columns (LC)	
ID	Method Description	ID	Method Description
BC-#1	The columns are simultaneously cut off	LC-#1	The columns are lifted simultaneously
BC-#2	The columns are cut off in chessboard style	LC-#2	The columns are lifted in multiple stages

Note: “BC” and “LC” represent “breaking columns” and “lifting columns”.

### 6.1. Disturbance of broken columns on structures

**Fig 18:**
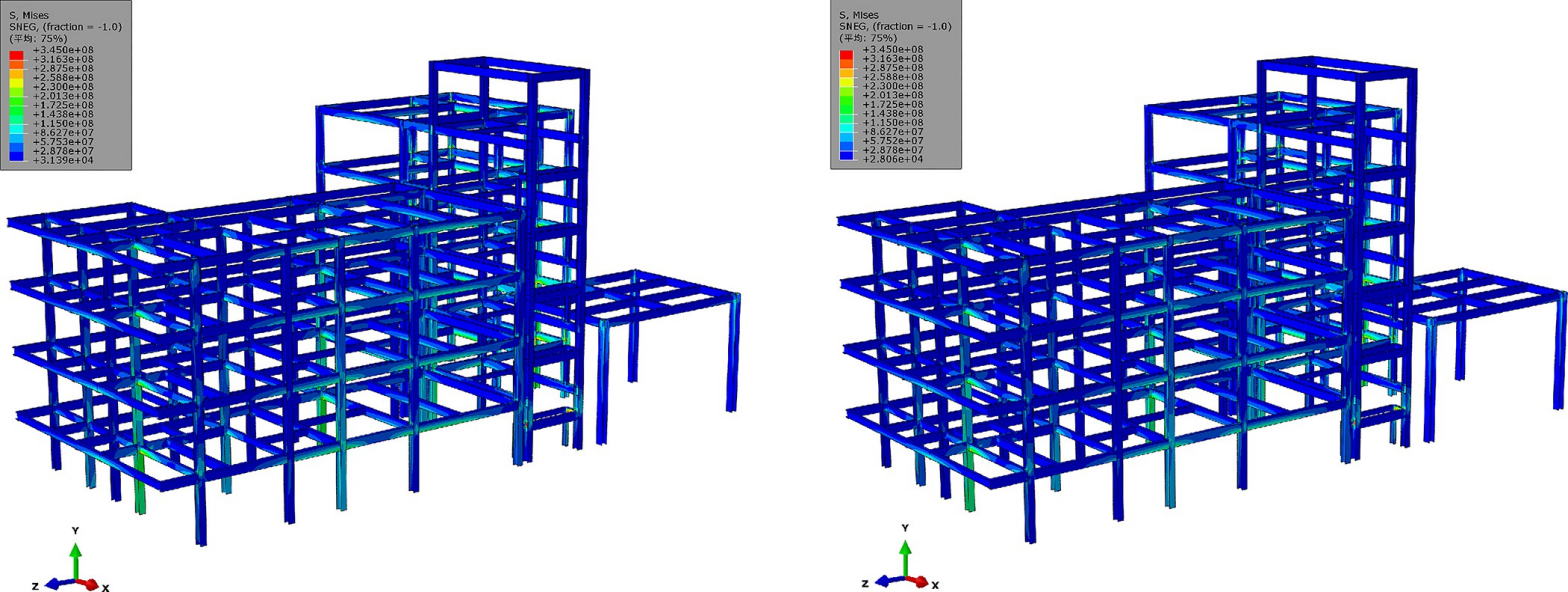
Von Mises stress field with different methods of broken columns (unit: Pa). (a) Von Mises stress field with BC-#1 (b) Von Mises stress field with BC-#2.

The calculated structural von Mises stress field with different breaking column (BC) methods is shown in Fig 18. For the analysis of the results, the final stage of structural response was checked, corresponding to the fifth stage in Fig 17. It can be observed that BC-#2 exhibits better stress release for the structure compared to BC-#1, especially in terms of stress release on joints and beams. Meanwhile, it is noted that after breaking columns using the BC-#1 method, the stress distribution along the marginal span is significantly higher than that of the BC-#2 method. There is also a noticeably larger stress distribution at the joints. The effect of BC methods on the stress distribution of floor slabs is relatively small, and the stress distribution patterns for both methods are essentially consistent. Regarding the development of structural von Mises stress, it is evident that the maximum von Mises stress for the material is 345 MPa, reaching the theoretical and experimental maximum yield stress values for Q345 structural steel. The maximum stress distribution is located at the outer columns of zone *F*, and there is a pronounced stress concentration phenomenon in the stairwell.

**Fig 19:**
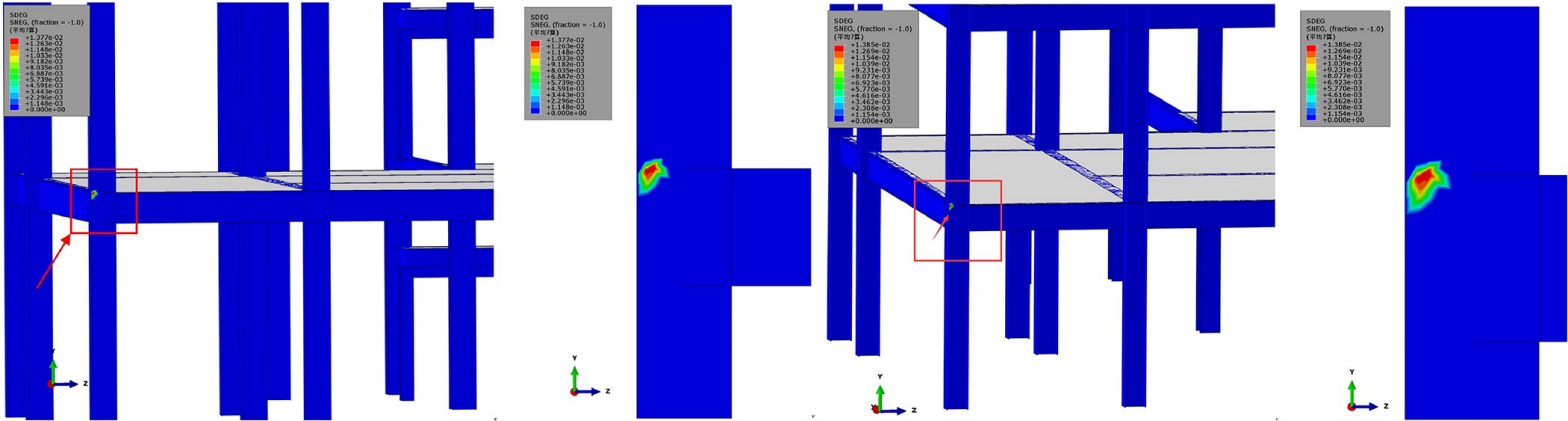
Damage zone with different inclination rectification methods (unit: Pa). (a) Joint damage with BC-#1; (b) Joint damage with BC-#2.

The damage and failure analysis of steel frame joints under two BCs methods is shown in Fig 19. It can be found that both methods exhibit a similar trend in the development of damage at the nodes. The comparative results indicate that the damage to the nodes primarily occurs at the first-floor node locations, with a maximum damage extent of approximately 0.01377 to 0.01385 (corresponding to a material stiffness reduction of 1.3%). The overall damage extent is relatively small. However, strengthening before uplifting at this location is still deemed necessary.

### 6.2. Structural response and evaluation after lifting columns

In the previous section, the impact of different breaking column methods on the structural stress field and local damage is investigated. The results indicate that the BC-#2 method induces smaller disturbances to the structure. Therefore, the effects of different uplifting methods on structural disturbances are compared in this section. The following analysis are based on the BC-#2 method for relevant discussions. This section will further explore the influence of different uplifting methods on the structure, namely LC-#1 and LC-#2 (refer to Table 6). The primary focus of the study is the distribution of the structural stress field after uplifting. By applying uplifting displacements to the FE model established in the previous section, the structural stress field is obtained to assess the damage and effects produced by different uplifting methods on the structure. The examination involves the stress disturbances on structural columns during the uplifting process, analyzing the degree of disturbance to the structural stress field caused by the two uplifting methods.

**Fig 20:**
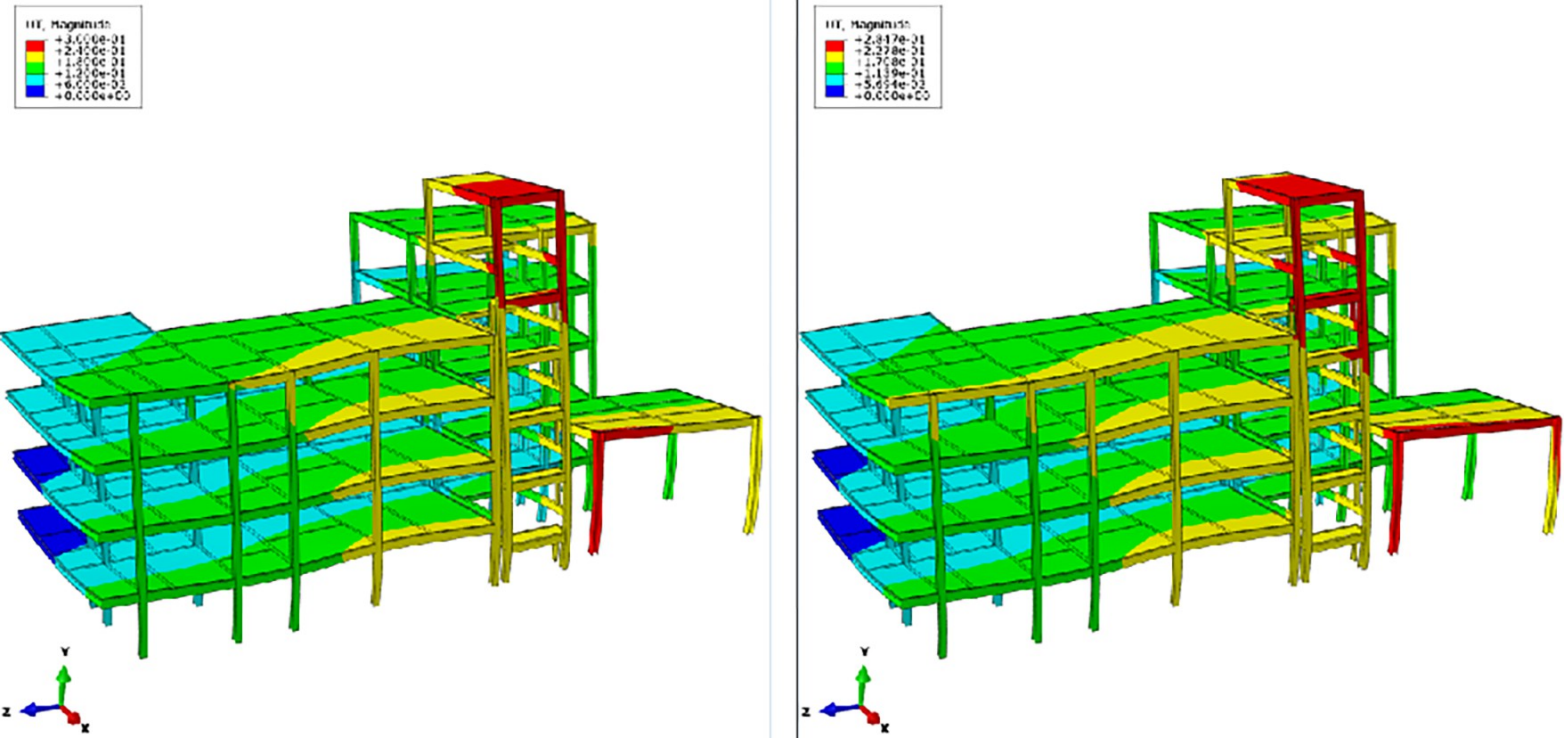
Structural deformation with different lifting methods (unit: M). (a) Structural deformation result with LC-#1 (Scale factor: 20);(b) Structural deformation result with LC-#2 (Scale factor: 20).

Fig 20 presents the maximum deformation contour maps of the structure after adopting two lifting methods, which including the steel frame and floor slabs, aiming to evaluate the overall deformation trends of the structure. It can be observed that, regardless of the two uplifting methods, the overall vertical deformation trends of the structure are basically consistent (Fig 20(A) and 20(B)). The lifting process does not significantly affect the overall deformation trends of the structure. However, concerning the maximum displacement contour map of the structure, there is a noticeable difference in the F zone’s single-layer shed under the two restoring techniques. In comparison to the LC-#2, LC-#1 exhibits significant lateral displacement diffusion in the outer columns of the single-layer shed, which implies that adopting the LC-#2 method can effectively reduce the influence between various structural columns and decrease the relative displacement between them.

**Fig 21:**
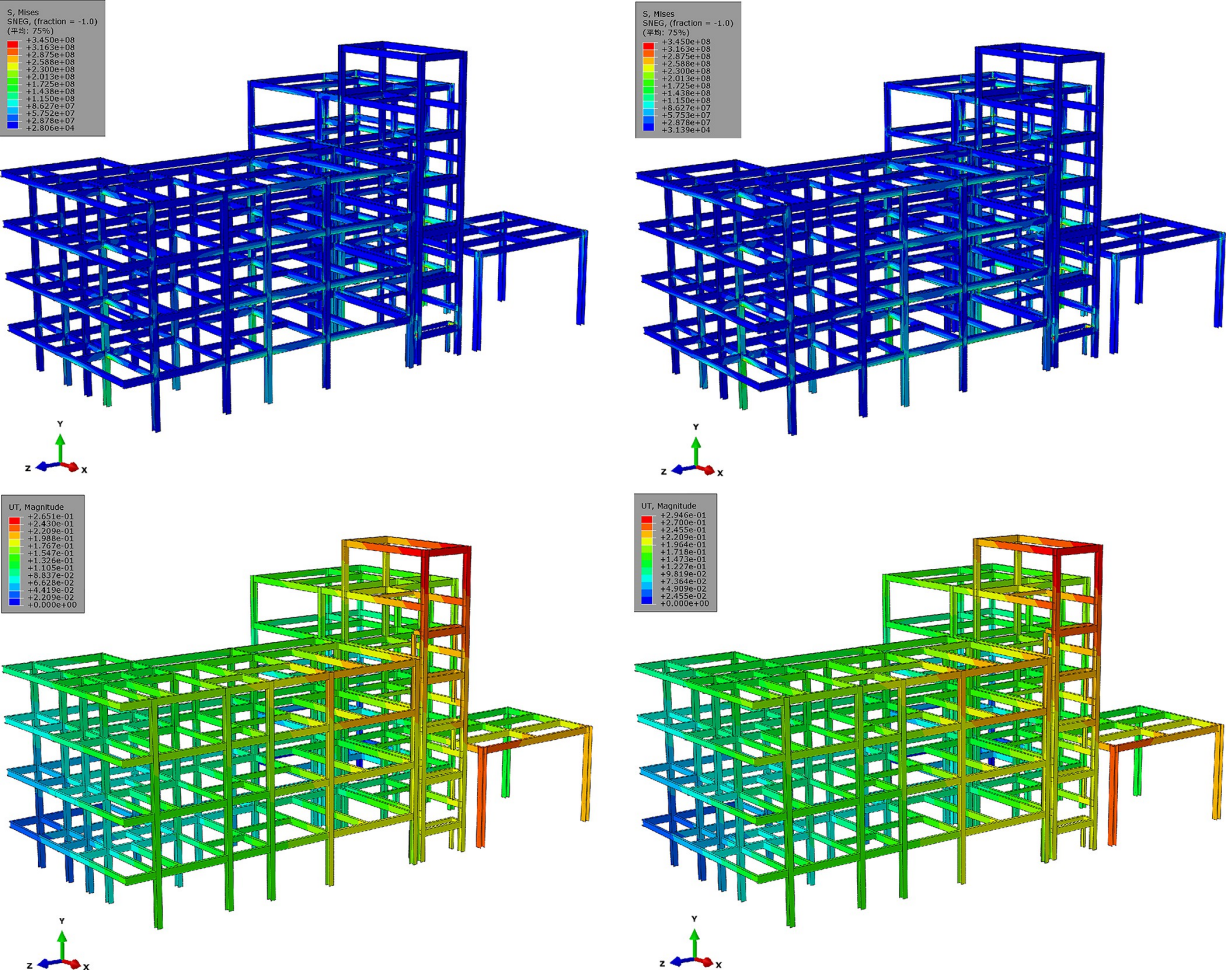
Structural mechanical response of steel frame with different lifting columns methods (unit: Pa and m). (a) Von Mises stress distribution with LC-#1;(b) Von Mises stress distribution with LC-#2;(c) Structural maximum displacement with LC-#1;(d) Structural maximum displacement with LC-#2.

Fig 21 presents the stress field and maximum displacement field of the structure after lifting with different methods, where the steel frame was emphasized for inspection. Upon comparing the stress field distributions, it is observed that the disparity in the impact of the two uplifting methods on the structure is within 5%. Regarding the influence on the displacement field, it is noted that the disturbance in the displacement field of LC-#2 is slightly smaller than that of LC-#1, with a maximum displacement difference of approximately 6%.

**Fig 22:**
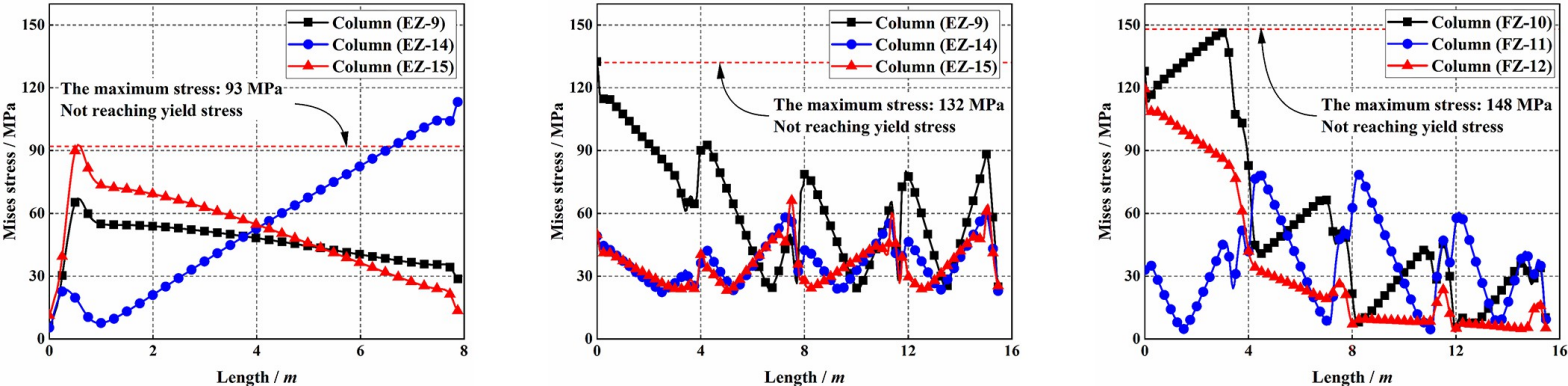
Von Mises stress distribution along the length direction of the column.

**Fig 23:**
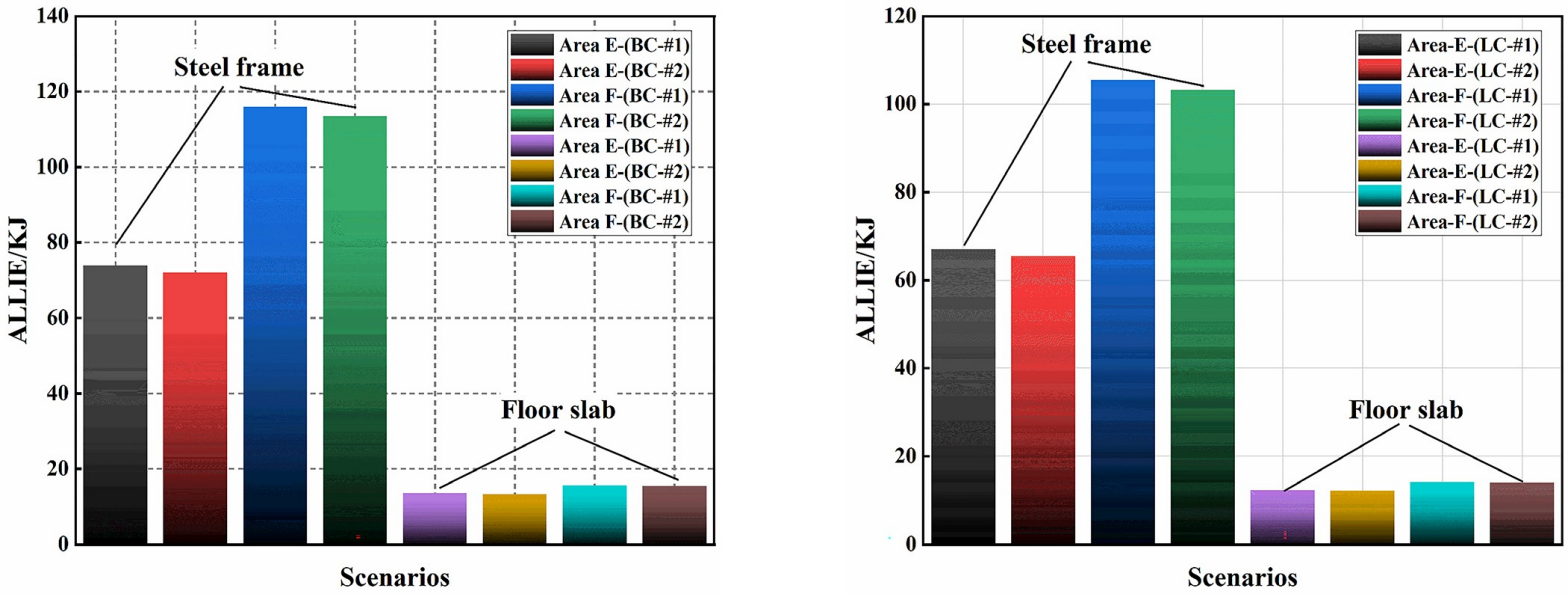
Energy dissipation with different methods.

Fig 22 illustrates the stress distribution along the height direction of structural key columns. It can be observed that apart from a small number of node positions where direct damage to the nodes occurs, no significant yielding behavior is detected on columns with higher stresses. The maximum stress variation also does not exceed the yield stress, and the stress disturbances on other structural columns are essentially negligible. Fig 23 presents the energy dissipation of the structure after uplifting under two different breaking and uplifting columns methods. It can be observed that LC-#2 and BC-#2 reduce the energy dissipation of the structure compared to LC-#1 and BC-#1, indicating lower stress and deformation disturbances. Additionally, their influence on the structure is smaller. However, overall, different schemes exhibit essentially the same trend in energy variation. The difference in energy dissipation for the structure between the two schemes does not exceed 10%.

Overall, this study presented four feasible methods for building correction. All four methods were capable of rectifying building lean, but they resulted in different levels of structural perturbations. BC-#1 and LC-#1 methods resulted in relatively larger structural damage after correction. The differences between the different methods primarily arisen from variations in the stress field disturbances and building damage during the building realignment process, attributed to different techniques. These differences mainly included additional stress on beams and columns, as well as damage to connections. Consequently, the selection of different methods should be determined by structural engineers based on engineering practicalities and economic considerations.

## 7. Conclusion

This study introduced the structural form and circumstances of an uneven settlement occurrence in a building based on an engineering case. Key steps during the correction processes, such as strengthening of column base (as shown in Fig 4), lateral force resistance strengthening of lower-level columns (as shown in Fig 5) and monitoring scheme (monitoring the joint damage with strain gauges, as shown in Fig 5) were comprehensively integrated. A design scheme and case for breaking column and lifting columns to rectify inclination was proposed. A refined FE model was established using FE simulation technology. The effects of different breaking columns methods and lifting columns methods on the corrective effectiveness and structural perturbations of buildings were evaluated. Four different parameters were provided and compared for each influencing factor. The following conclusions were drawn:

1. This study provided a detailed description of the processes and methods of breaking columns and lifting columns after the occurrence of uneven settlement in a building. The proposed scheme effectively corrects the building and ensures the integrity and safety of its structure.
2. A refined FE model of the correction area in the building was established using ABAQUS numerical simulation software. The accurate material constitutive behavior was adopted with detailed damage mechanics models applied to concrete, beams and columns. These models were capable of examining the structural damage conditions after settlement, breaking columns and lifting columns, providing targeted guidance for engineering practice and subsequent strengthening work.
3. The impact of uneven settlement on structural disturbances was analyzed. Modal characteristic results indicated a significant reduction in structural eigenvalues and natural frequencies after settlement, significantly affecting the overall stability and safety of the structure. The structural stress field experienced considerable disturbance after uneven settlement, with materials entering the yielding stage at some joints, resulting in material damage. Although the material has not reached the complete failure stage, the structural safety was significantly compromised.
4. The overall displacement, stress disturbances and damage states of the structure after adopting two breaking column methods were analyzed. The BC-#2 and BC-#1 were compared. Regarding stress field disturbances and structural stability, BC-#2 demonstrated a more noticeable advantage. Two lifting column methods, LC-#1 and LC-#2, both successfully corrected the structure. However, LC-#2 has a lesser impact on the structure from the perspective of structural disturbances.
5. In summary, it was recommended to use BC-#2 and LC-#2 for the correction of uneven settlement in light steel-steel frame structures. This approach proved more effective in reducing structural disturbances, avoiding damage and minimizing the impact on the initial structure.

Note: Currently, the correction methods proposed in the study have demonstrated better performance and less structural perturbations in steel structures. The main challenges of these methods lay in how to avoid the potential structural damage during the correction process for different structural styles even if the same method was used, including damage to connections and disturbances to the initial structure. Therefore, it was recommended that structural engineers adhered to the modeling approach proposed in this study and conduct inspections of the correction schemes to ensure minimized structural disturbances.

## Supporting information

S1 File (ZIP)
